# Nanoscale Buried Interface Control in Silicone Hydrogels Enables a Biointerface Engineering Platform

**DOI:** 10.1002/advs.78148

**Published:** 2026-10-19

**Authors:** Po‐Hsiang Chen, Jia‐Hua Wang, Ching Chen, Shih‐Hsuan Fu, Rajan Deepan Chakravarthy, Hsin‐Chieh Lin

**Affiliations:** ^1^ Department of Materials Science and Engineering National Yang Ming Chiao Tung University Hsinchu Taiwan (ROC); ^2^ Center for Intelligent Drug Systems and Smart Bio‐devices (IDS^2^B) National Yang Ming Chiao Tung University Hsinchu Taiwan (ROC)

**Keywords:** biointerface engineering, hydrogel, multimodal characterization, nanocoatings, semi‐interpenetrating polymer network

## Abstract

Engineering multifunctional biointerfaces requires quantitative understanding of how buried nanoscale coating architectures govern biological function, yet such relationships remain inaccessible because non‐destructive characterization of soft, curved interfaces is lacking. Here, we establish an integrated engineering–characterization–function platform that quantitatively resolves buried nanocoatings and directly correlates their architecture with interfacial performance. Using silicone hydrogel contact lenses as a representative biomedical substrate, robust semi‐interpenetrating polymer network (semi‐IPN) nanocoatings are fabricated and characterized by gas cluster ion beam X‐ray photoelectron spectroscopy (GCIB‐XPS), time‐of‐flight secondary ion mass spectrometry (ToF‐SIMS), and a newly developed atomic force microscopy (AFM) modulus‐based algorithm. This multimodal framework non‐destructively maps vertical chemical gradients, nanometer‐scale layer thicknesses, and interfacial mechanical distributions with sub‐100 nm precision, enabling quantitative visualization of conformal buried nanocoatings. The resulting structure–function relationships guide the rational engineering of poly(2‐hydroxyethyl methacrylate) (poly(HEMA)) interfaces with homogeneous surface coverage (∼89%), enhanced wettability, markedly reduced protein adsorption and cellular fouling, while fully preserving oxygen permeability, optical transparency, UV protection, and mechanical integrity. The platform further enables incorporation of functional monomers for light‐triggered therapeutic release without compromising device performance. Beyond contact lenses, this work provides a generalizable strategy for quantitatively engineering and predicting multifunctional nanocoatings across diverse biomedical interfaces.

## Graphical abstract

A hydrophilic coating with controllable nanoscale thickness is applied to commercial silicone hydrogel contact lenses. By combining comprehensive multi‐modal three‐dimensional characterization with an advanced algorithm, the thickness and chemical information of these multilayered structures are resolved. This universal platform enhances surface wettability and resists biofouling, seamlessly enabling the development of smart, light‐responsive ophthalmic drug delivery bio‐interfaces.

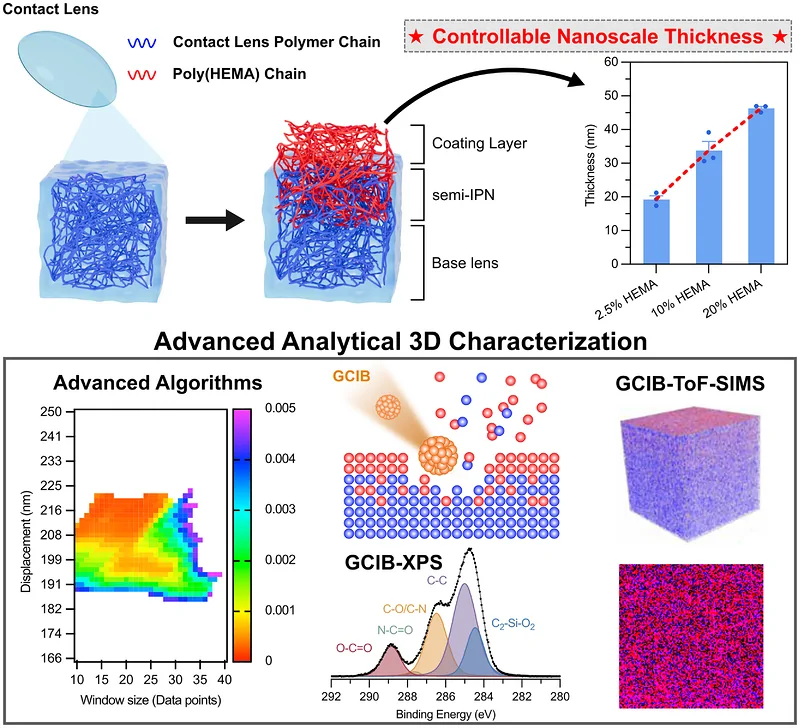

## 1 Introduction

Surface engineering has become a central strategy for tailoring the biological performance of medical devices, enabling diverse functions including antifouling, lubrication, controlled drug delivery, and tissue integration [1, 2, 3, 4, 5, 6, 7]. Specific applications include applying zwitterionic polymer brushes to modify blood‐contacting catheters and prevent thrombosis [8], utilizing layer‐by‐layer (LbL) assembly to encapsulate therapeutics on microneedles for controlled release [9], employing polyethylene glycol (PEG) to protect implantable sensors against biofouling and foreign body responses [10], and incorporating polydopamine (PDA) to enhance surface wettability and promote cell adhesion on tissue scaffolds and drug‐eluting implants [11, 12]. The success of these functional coatings depends not only on their chemical composition but also on their nanoscale interfacial architecture, which governs interactions between synthetic materials and biological environments. Soft hydrogel‐based devices present a particularly stringent model system because their hydrated, compliant, and curved interfaces make both coating engineering and quantitative characterization exceptionally challenging. Among these, silicone hydrogel contact lenses provide an ideal model system because they combine a soft, hydrated, oxygen‐permeable bulk with a chemically heterogeneous surface [13, 14]. Although modern silicone hydrogels possess excellent bulk oxygen permeability, the hydrophobic silicone domains inevitably segregate toward the interface in aqueous environments [15, 16, 17], resulting in poor wettability, protein adsorption, tear‐film instability, and increased friction [18, 19, 20, 21]. Consequently, extensive efforts have focused on engineering hydrophilic surface coatings while preserving the intrinsic bulk properties of the substrate.

Despite remarkable advances in coating chemistries, a fundamental challenge remains unresolved. The biological performance of biomedical coatings is fundamentally governed by their three‐dimensional (3D) interfacial architecture, including nanoscale thickness, chemical composition, spatial distribution, and mechanical heterogeneity. Yet, these critical structural attributes remain largely inaccessible to quantitative characterization on soft, curved, and hydrated substrates. Existing coating technologies primarily emphasize fabrication, whereas their characterization relies on conventional techniques such as Raman spectroscopy [22], fluorescence imaging [23], or atomic force microscopy (AFM) [24], each providing only partial structural information and being largely limited to planar surfaces. As a result, it remains extremely difficult to quantitatively determine how coating architecture governs biological performance, preventing the rational design of multifunctional biointerfaces.

The inability to establish quantitative structure–function relationships represents a fundamental bottleneck that extends far beyond contact lenses. Numerous biomedical devices—including intraocular lenses, catheters, microneedles, implantable sensors, tissue scaffolds, and drug‐eluting implants—depend on conformal nanometer‐scale coatings to regulate interfacial biological interactions on complex three‐dimensional geometries. However, current analytical methods are incapable of simultaneously resolving the chemical composition, nanoscale thickness, interfacial mechanics, and spatial distribution of these multilayered coatings in a quantitative and non‐destructive manner. Consequently, coating optimization continues to rely predominantly on empirical trial‐and‐error rather than predictive interfacial engineering.

Here, we establish an integrated engineering–characterization–function framework for multifunctional biointerfaces. Using silicone hydrogel contact lenses as a representative curved biomedical substrate, we construct a robust semi‐interpenetrating polymer network (semi‐IPN) through the physical entanglement of poly(2‐hydroxyethyl methacrylate) (poly(HEMA)) within the subsurface silicone matrix. More importantly, we develop a multimodal characterization platform integrating gas cluster ion beam X‐ray photoelectron spectroscopy (GCIB‐XPS), time‐of‐flight secondary ion mass spectrometry (ToF‐SIMS), and a newly developed AFM modulus‐based computational algorithm. This integrated framework enables, for the first time, quantitative visualization of vertical chemical gradients, nanometer‐scale layer thicknesses, and interfacial mechanical distributions within buried multilayer coatings on curved hydrogel substrates.

By quantitatively correlating coating chemistry, nanoscale thickness, mechanical architecture, and biological function, we demonstrate that the engineered semi‐IPN interface effectively decouples surface wettability from bulk oxygen permeability while simultaneously suppressing biofouling and preserving optical transparency, mechanical integrity, and clinical UV safety. Finally, we extend this platform by incorporating a light‐responsive prodrug, demonstrating that precise nanoscale architectural control enables predictable therapeutic release without compromising device performance. Beyond silicone hydrogel contact lenses, this integrated platform provides a general framework for engineering and quantitatively characterizing multifunctional nanocoatings across a broad range of curved biomedical interfaces.

## 2 Results and Discussion

### 2.1 Design of Modification Strategy and 3D Characterization of the Modified Materials

To establish a quantitative structure–function relationship between nanoscale coating architecture and biointerface performance, we developed a representative curved hydrogel model system based on commercially available silicone hydrogel contact lenses. In this study, we investigated three commercially available silicone hydrogel contact lenses: Acuvue Oasys (Senofilcon A; Johnson & Johnson), Clariti 1 day (Somofilcon A; CooperVision), and Ultra One Day (Kalifilcon A; Bausch + Lomb). For brevity, these lenses are referred to as Acuvue, Clariti, and Ultra, respectively. To bridge the gap between the high oxygen permeability of silicone hydrogels and the superior surface hydrophilicity and biocompatibility of conventional hydrogels, we engineered a hybrid interface by grafting a hydrophilic poly(HEMA) layer onto the silicone hydrogel substrates (Figure 1a). This strategy aims to create a novel contact lens where the core preserves high oxygen flux while the surface displays the favorable attributes of hydrophilic hydrogel materials.

**FIGURE 1:**
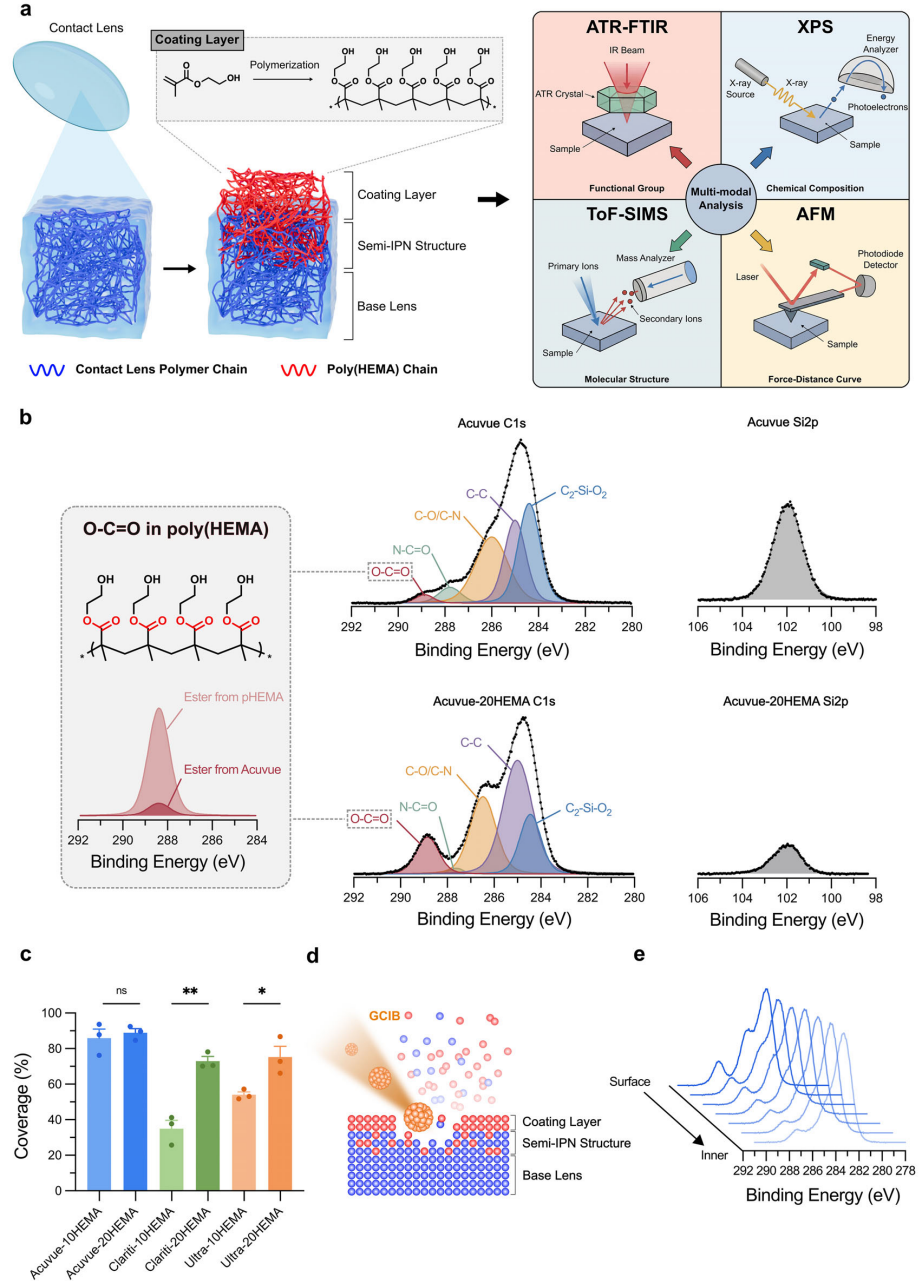
Characterization of HEMA‐modified contact lenses. (a) Schematic illustration of HEMA polymerization on a commercial contact lens surface. The resulting poly(HEMA) chains (red) entangle with the polymer network of the base lens (blue) to form a poly(HEMA) coating layer and semi‐IPN structure. (b) High‐resolution XPS C1s and Si2p spectra of pristine Acuvue and Acuvue‐20HEMA. The deconvoluted C1s spectra reveal the emergence of the ester functionality (O─C═O, red peak) derived from the poly(HEMA) coating. (c) Calculated poly(HEMA) surface coverage based on XPS atomic concentration data. (d,e) Depth profiling analysis. Schematic of the GCIB sputtering process (d) and the corresponding evolution of high‐resolution C1s XPS spectra at progressive sputtering depths (e). The waterfall plot shows the gradual attenuation of the ester peak from the surface to the lens interior (5 min sputtering intervals). Data in c are presented as mean ± s.e.m. (*n* = 3). Statistical significance was calculated using *t‐*test: ns, not significant (*p* > 0.05), *p < 0.05, **p < 0.01. The schematics in panels (a) and (d) were prepared using Inkscape; 3D elements in panel (a) were modeled and rendered in Blender. Selected visual elements in panel (a) were generated using Gemini Nano Banana 2 and subsequently edited and integrated in Inkscape. All scientific content was reviewed and verified by the authors.

Under our experimental conditions, the pristine contact lens serves as the pre‐existing crosslinked network within the semi‐IPN architecture. Initially, the pre‐existing crosslinked matrix is swollen to absorb the VA‐086 initiator, then immediately transferred into the HEMA‐saturated solution. Upon radical initiation, the absorbed initiator begins to propagate with the surrounding HEMA monomers. This polymerization process generates numerous linear polymer chains that are intricately entangled within the foundational crosslinked matrix, thereby successfully forming the semi‐IPN structure. The confinement of the VA‐086 initiator strictly to the lens surface, avoiding deep bulk penetration, is governed by a combination of thermodynamic incompatibility, interfacial molecular interactions, and precise kinetic control. First, 2,2'‐azobis[2‐methyl‐N‐(2‐hydroxyethyl)propionamide] (VA‐086) is a highly hydrophilic initiator. Because the silicone hydrogel lens contains a substantial hydrophobic phase composed of siloxane segments, this inherent hydrophilic‐hydrophobic incompatibility serves as a primary thermodynamic barrier that prevents VA‐086 from permeating deeply into the lens bulk. Second, during swelling, the hydroxyl and amide groups on the VA‐086 molecules readily form strong intermolecular hydrogen bonds with the hydrophilic domains on the lens surface, including the polymerized *N,N*‐dimethylacrylamide (DMAA), *N*‐vinylpyrrolidone (NVP), and HEMA components. This strong hydrogen bonding network effectively anchors the initiator at the interface, severely restricting its inward diffusion kinetics. Finally, in our experimental design, we kinetically restricted penetration by limiting the VA‐086 immersion time to 30 min. This short timeframe ensures that VA‐086 only permeates the outermost superficial region of the lens during the swelling phase.

The modified samples were prepared according to HEMA concentration and were designated as Acuvue‐2.5HEMA, Acuvue‐10HEMA, Acuvue‐20HEMA, Clariti‐2.5HEMA, Clariti‐10HEMA, Clariti‐20HEMA, Ultra‐2.5HEMA, Ultra‐10HEMA, and Ultra‐20HEMA. Commercially available Acuvue, Clariti, and Ultra lenses were used as controls to explore the relationship between HEMA concentration and contact lens performance. This experimental design provides a quantitative platform for systematically investigating how coating chemistry and nanoscale architecture influence interfacial properties, biological responses, and therapeutic functionality, thereby establishing the structure–function relationships underlying multifunctional biointerface engineering.

To elucidate the 3D spatial distribution of poly(HEMA) on the silicone hydrogel surface, we employed a suite of advanced analytical characterization techniques, including attenuated total reflectance Fourier‐transform infrared spectroscopy (ATR‐FTIR), XPS, ToF‐SIMS, and AFM (Figure 1a). Initial ATR‐FTIR analysis provided a rapid, qualitative assessment of surface functional groups. All three types of HEMA‐modified lenses (10% and 20% HEMA) exhibited intensified absorption bands at 1,158 cm^−1^ (C─O─C) and 1,720 cm^−1^ (C═O), confirming the presence of the ether and ester functional groups of the poly(HEMA) chains, respectively [25]. The broad O‐H stretching vibration (3,300–3,500 cm^−1^) also increased with higher HEMA monomer concentrations (Figure S1). However, the penetration depth (*d*_e_) of the diamond ATR crystal is limited to approximately 3–6 µm at 1,000 cm^−1^, probing the bulk‐like region rather than the immediate surface interface [26].

To resolve the composition of the outermost layer with higher specificity, we performed XPS, which probes the top ∼10 nm of the surface [27]. Elemental survey spectra for three pristine control lenses revealed carbon, nitrogen, oxygen, and silicon as the principal elements (Figure S2). The carbon (C) ratio showed minimal variation before and after the coating, as both the pristine contact lenses and the poly(HEMA) layer share a predominantly carbon‐based backbone. In contrast, the oxygen (O) content consistently increased across all three lens types following modification. Specifically, these values rose from 21.21% ± 0.88% to 28.15% ± 0.56% for Acuvue, from 18.44% ± 0.13% to 23.37% ± 0.76% for Clariti, and from 18.43% ± 0.99% to 22.33% ± 1.35% for Ultra. This upward trend is directly attributed to the high oxygen density of the HEMA monomer, which contains three oxygen atoms. Conversely, the detection of silicon (Si) and nitrogen (N) originates from the siloxane backbone and functional monomers (e.g., DMAA or NVP) of the base lens. The atomic ratios of these substrate‐specific elements consistently decreased following modification. Notably, both Si2p and N1s intensities progressively declined with increasing HEMA concentration, indicating the effective masking of the underlying silicone substrate by the growing poly(HEMA) overlayer (Table S1 and Figures S2 and S3).

To obtain precise compositional maps, we analyzed high‐resolution XPS spectra of all lenses (Figure 1b and Figure S4). All HEMA‐modified lenses displayed a pronounced peak at 288.5 eV, corresponding to the ester group (O─C═O) of poly(HEMA). This signal was significantly intensified in Acuvue‐20HEMA compared to the pristine Acuvue control, while the substrate‐specific signals (C_2_‐Si‐O_2_ and Si2p) were concurrently suppressed, validating the effective masking of the silicone hydrogel by the poly(HEMA) overlayer (Figure 1b). We quantified the poly(HEMA) surface coverage by normalizing the ester component of the C1s envelope to the substrate silicon signal (Equations (1) and (2)). Both Acuvue‐10HEMA and Acuvue‐20HEMA achieved the highest surface coverage, reaching 85.87% ± 5.10% and 88.92% ± 2.27%, respectively, significantly outperforming the HEMA‐modified (10% and 20% HEMA) Clariti and Ultra lenses, ranging from 34.90% ± 4.70% to 75.24% ± 6.06% (Figure 1c). This superior coverage is attributed to the substrate's unique swelling characteristics. Specifically, Acuvue exhibits the lowest swelling ratio (47.2% ± 1.0%) compared to Clariti (104.4% ± 1.1%) and Ultra (98.2% ± 1.9%) (Table 1). This restricted swelling environment facilitates the growth of longer poly(HEMA) chains, which enhances the masking efficiency of the underlying substrate, thereby resulting in the highest observed surface coverage (Note S1). Significantly, this near‐saturation coverage (∼89%) represents a substantial advancement over conventional surface functionalization strategies reported in the literature, drastically outperforming the coverages typically achieved via click chemistry (10% and 33%) [28, 29] or atom transfer radical polymerization (ATRP, 44%) [30] (Notes S2–S4).

**TABLE 1:** Comprehensive summary of the structural, physicochemical, and biological properties of pristine and HEMA‐modified contact lenses.

	Surface Coverage (%)	Layer thickness (nm)		Static Contact Angle (°)	Swelling Ratio (%)	Roughness, *R*_q_ (nm)	BSA Adsorption (µg/cm^2^)	Lysozyme Relative Activity (%)	Cell Adhesion Ratio (%)
		Coating	Semi‐IPN						
Acuvue	—	—	—	90.31 ± 0.67	47.2 ± 1.0	4.60 ± 0.07	20.80 ± 0.69	10.61 ± 1.42	63.02 ± 1.67
2.5 HEMA	—	19.23 ± 1.10	—	—	—	—	—	—	—
10 HEMA	85.87 ± 5.10	33.75 ± 2.71	25.18 ± 2.74	54.76 ± 1.12	—	—	14.36 ± 0.37	13.24 ± 1.60	58.37 ± 1.24
20 HEMA	88.92 ± 2.27	46.28 ± 0.61	70.26 ± 1.60	55.64 ± 0.85	—	2.67 ± 0.08	11.49 ± 0.30	29.74 ± 3.84	54.62 ± 0.11
Clariti	—	—	—	88.88 ± 0.41	104.1 ± 1.1	4.40 ± 0.09	48.03 ± 0.18	8.76 ± 0.73	64.84 ± 0.94
2.5 HEMA	—	36.83 ± 5.31	—	—	—	—	—	—	—
10 HEMA	34.90 ± 4.70	46.22 ± 3.66	25.44 ± 3.65	73.30 ± 0.49	—	—	42.80 ± 1.06	14.98 ± 1.60	55.45 ± 1.05
20 HEMA	72.96 ± 2.62	71.68 ± 6.74	74.39 ± 5.70	65.06 ± 1.36	—	3.53 ± 0.18	19.16 ± 1.47	29.58 ± 1.79	55.38 ± 0.14
Ultra	—	—	—	87.19 ± 0.48	98.2 ± 1.9	4.63 ± 0.27	39.31 ± 0.64	11.02 ± 2.13	69.70 ± 0.33
2.5 HEMA	—	39.60 ± 6.47	—	—	—	—	—	—	—
10 HEMA	54.00 ± 1.64	36.74 ± 0.58	22.62 ± 2.20	77.16 ± 0.50	—	—	33.74 ± 1.09	22.23 ± 1.45	61.80 ± 1.71
20 HEMA	75.24 ± 6.06	54.31 ± 2.00	67.38 ± 3.93	67.16 ± 0.88	—	3.57 ± 0.04	31.34 ± 0.40	30.23 ± 3.45	60.31 ± 1.30

While standard XPS is surface‐limited (∼10 nm), probing the buried interface is essential to confirm the semi‐IPN architecture. We utilized GCIB sputtering to perform damage‐free organic depth profiling (Figure 1d,e and Figure S6) [31, 32]. The intensity of the characteristic ester peak (288.5 eV) exhibited a gradual attenuation with increasing sputtering depth rather than an abrupt cutoff. This gradient profile indicates that the poly(HEMA) is not merely a distinct superficial layer but is chemically graded into the underlying base lens (Figure S7).

While GCIB depth profiling confirms the chemical gradient, it is intrinsically limited in its ability to precisely quantify the physical thickness of the discrete coating and semi‐IPN layers. To resolve this architecture with nanometric precision, we characterized the interface using AFM in quantitative nanomechanical mapping (QNM) mode (Figure 2a). As a key feature, unlike vacuum‐based spectroscopic techniques (e.g., XPS and ToF‐SIMS), AFM operates in an aqueous environment, allowing us to characterize the materials in their fully hydrated, physiologically relevant state, thereby capturing the most authentic representation of the operational bio‐interface.

**FIGURE 2:**
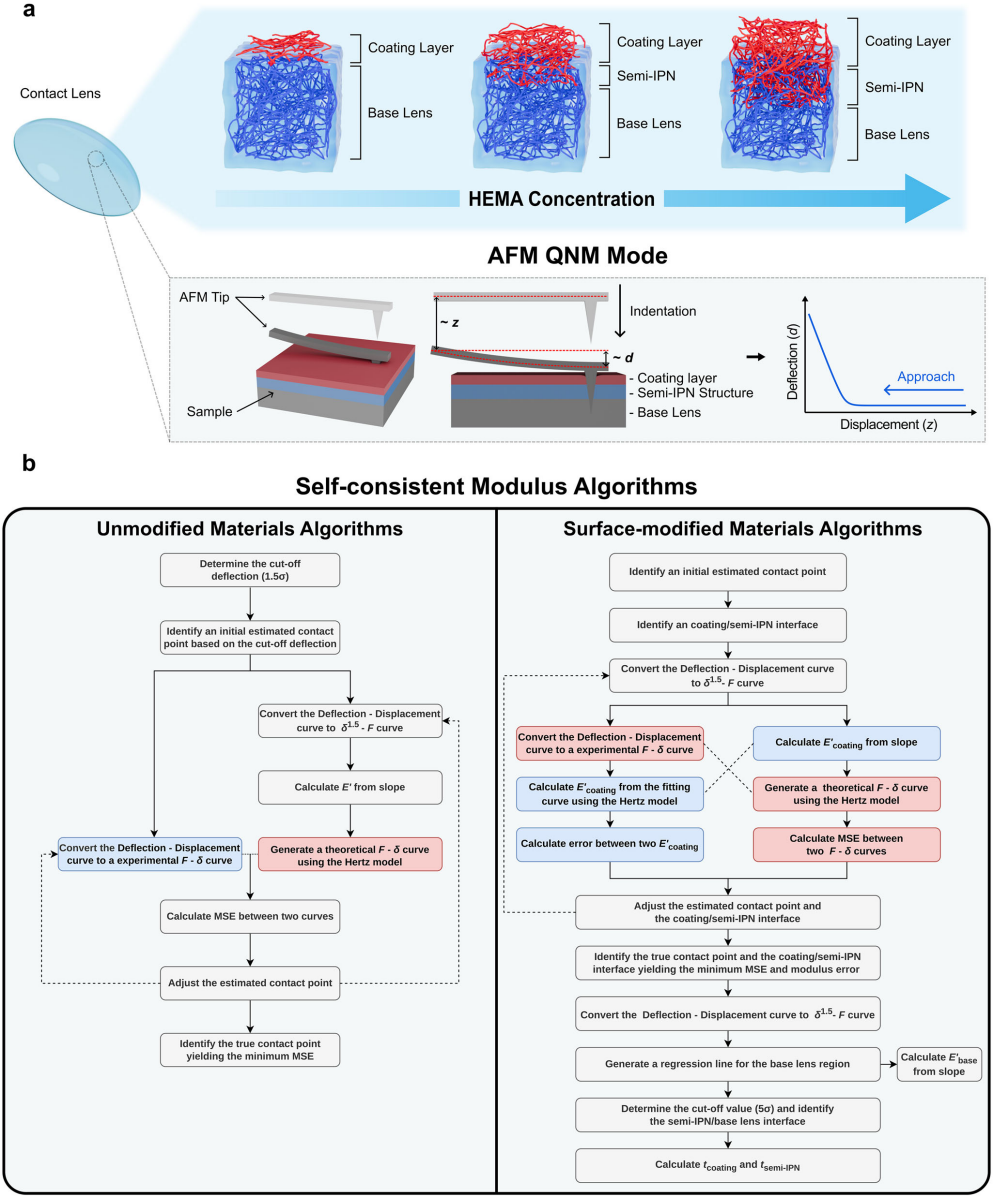
Overview of structural evolution and nanomechanical analysis. (a) Schematic illustrating the concentration‐dependent evolution of the coating layer and semi‐IPN with increasing HEMA monomer concentration. The lower panel depicts the AFM QNM indentation process on the modified lens, where *z* and *d* denote tip displacement and cantilever deflection, respectively. (b) Flowcharts of the algorithms used to analyze the pristine and HEMA‐modified lenses. The schematic in panel (a) was prepared using Inkscape; 3D elements were modeled and rendered in Blender.

By exploiting the mechanical contrast across the interface, we successfully differentiated the structural layers. The rigid base lens exhibits the highest modulus, whereas the fully hydrated hydrophilic coating yields the lowest. Notably, the semi‐IPN structure displays an intermediate stiffness, acting as a continuous mechanical transition characteristic of the entangled polymer network.

Capitalizing on the high vertical resolution of AFM, we extended our analysis to include a 2.5% HEMA group alongside the standard 10% and 20% formulations. This allowed us to explicitly verify that the thicknesses of both the poly(HEMA) layer and the semi‐IPN structure can be precisely modulated by varying the monomer concentration.

We developed a custom self‐consistent modulus algorithm based on the Hertz model to extract quantitative thickness data from force‐distance curves (FDCs) (Figure 2b). For all three types of pristine control lenses, the algorithm identifies the true surface contact point (red point) by minimizing the mean squared error (MSE) between experimental data and theoretical fits (Figure 3a–d and Figures S9 and S10).

**FIGURE 3:**
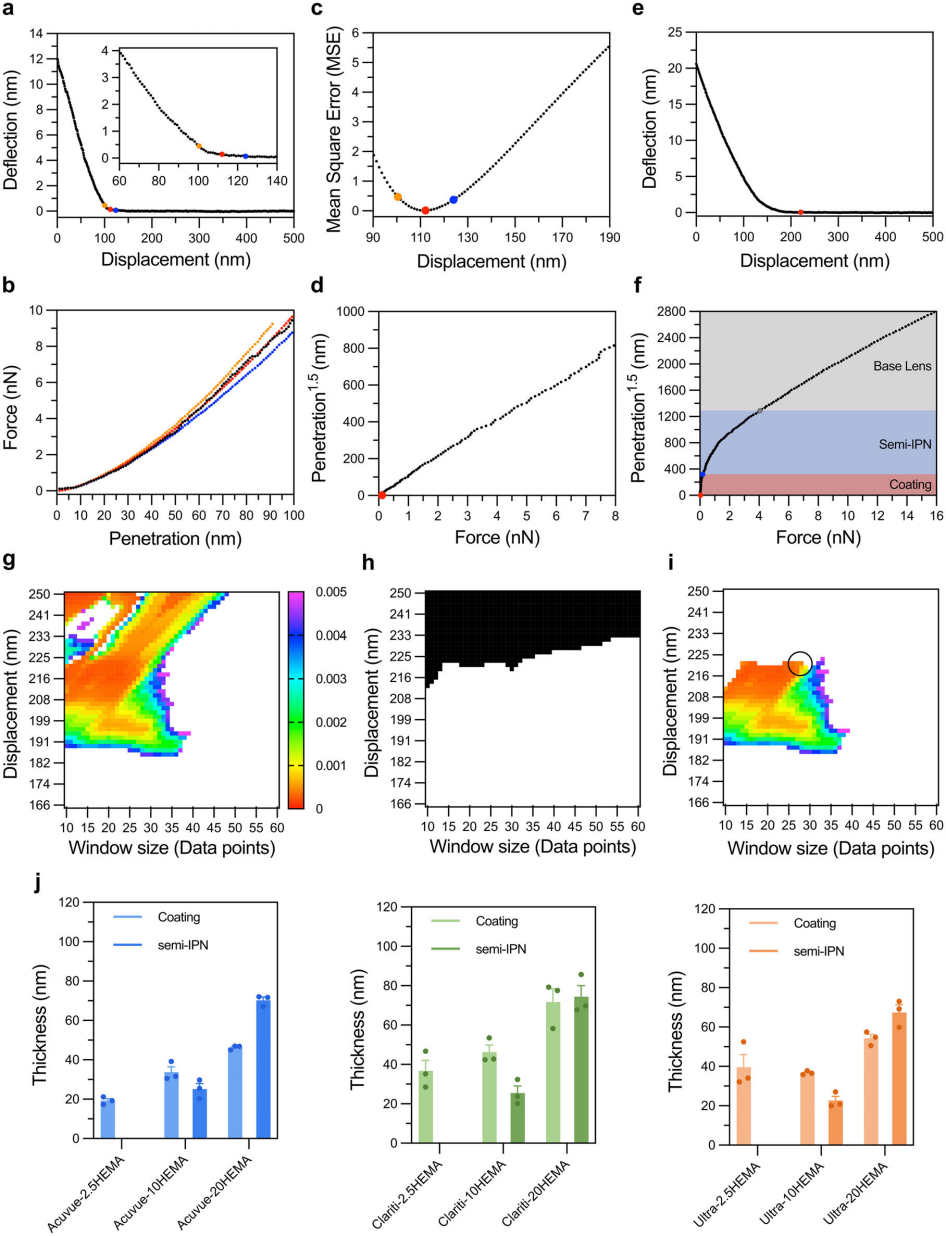
Thickness analysis of HEMA‐modified layers via self‐consistent modulus algorithms. (a–d) Algorithm validation on a pristine Acuvue lens. Representative deflection curve (a) showing the true contact point (red) relative to estimated deviations (blue, orange). The accuracy of the contact point determination is verified by comparing experimental and theoretical force–distance curves (FDCs) (b) and minimizing the mean squared error (MSE) (c). The reduced modulus (*E'*) is derived from the slope of the linearized force–indentation plot (d). (e–i) Analysis of the multilayered Acuvue‐20HEMA interface. The deflection curve (e) and linearized indentation plot (f) reveal three distinct mechanical regimes: the outer coating (red), the semi‐IPN structure (blue), and the base lens (grey). The optimal interface location is identified by mapping the MSE (g) and modulus error (h) across varying window sizes and displacements. In (h), black regions denote a modulus error > 15%, whereas transparent regions indicate an error ≤ 15%. The combined heatmap (i) pinpoints the global solution (black circle) that minimizes MSE within the valid modulus error range (≤ 15%). (j) Quantified thicknesses of the coating layer and semi‐IPN structure for Acuvue, Clariti, and Ultra lenses modified with 2.5%, 10%, and 20% HEMA. Data are presented as the mean ± s.e.m. (*n* = 3).

For all three types of HEMA‐modified lenses (2.5%, 10% and 20% concentration), the algorithm was extended to identify two critical transitions: the surface contact point (red point) and the coating/semi‐IPN interface (blue point) (Figure 3e,f and Figures S8–S10). A two‐dimensional stacked heatmap facilitated the global optimization of these parameters (Figure 3g–i). This analysis clearly demarcated three distinct mechanical regimes: the outer poly(HEMA) coating (red region), the intermediate semi‐IPN structure (blue region), and the underlying base lens (gray region). Unlike conventional AFM methodologies that are restricted to identifying a single surface contact point on homogenous materials [33], our algorithm successfully resolves the internal interfaces of complex multi‐layered architectures. Furthermore, while traditional characterization techniques are often limited to micrometer‐scale resolution [22, 23], this approach enables quantitative depth profiling with nanometric precision.

This methodology revealed concentration‐dependent growth kinetics. Specifically, the thicknesses of both the pure coating layer and the semi‐IPN structure increased progressively as the HEMA concentration was elevated from 2.5% to 20%, demonstrating that the vertical dimension of the modification can be precisely modulated. (Figure 3j). Notably, HEMA‐modified Clariti lenses demonstrated significantly greater poly(HEMA) layer thicknesses (ranging from 36.83 ± 5.31 to 71.68 ± 6.74 nm) compared to HEMA‐modified Acuvue (19.23 ± 1.10–46.28 ± 0.61 nm) and Ultra (39.60 ± 6.47–54.31 ± 2.00 nm) lenses (Figure 3j), a finding that correlates with their intrinsic swelling ratios. Specifically, the swelling ratio of Clariti (104.4% ± 1.1%) is markedly higher than that of Ultra (98.2% ± 1.9%) and Acuvue (47.2% ± 1.0%), which likely facilitates a higher adsorption of the initiator, thereby accelerating the polymerization rate (Table 1 and Table S2 and Note S1). Significantly, this capability to tune the modification layer at the nanometer scale represents a distinct advantage over commercial water gradient technologies. While current water gradient platforms are restricted to micrometer‐scale surface layers, maintaining thicknesses of approximately 6 µm for Delefilcon A and 2–3 µm for Verofilcon A, our approach achieves highly precise, nanometric interfacial engineering [34, 35].

This architecture offers a distinct advantage over conventional plasma treatments, which are typically restricted to ultrathin surface modifications (∼20 nm) and lack a penetrating network [24]. In contrast, our approach generates a semi‐IPN structure that is firmly anchored to the lens surface via physical entanglement. The stability of the coating was rigorously evaluated through an extensive series of durability tests (Figure S11). These comprehensive evaluations encompassed long‐term storage in aqueous media, physiological saline, and simulated tear fluid at physiological temperatures, repeated hydration/dehydration cycles, simulated mechanical wear from blinking, and accelerated aging. The structural stability of the coating was systematically monitored via static water contact angle measurements. Following a prolonged 14‐day storage period across all tested liquid media, the contact angle data clearly demonstrated that all three types of HEMA‐modified lenses successfully retained their exceptional hydrophilic properties. This extended storage evaluation effectively alleviates concerns regarding the potential hydrolysis of the poly(HEMA) coating. Although HEMA is susceptible to hydrolysis under strong acidic or basic conditions (for instance, undergoing approximately 80% degradation after 14 days at a pH below 2) [36], it remains chemically stable and highly resistant to hydrolysis under standard physiological conditions. Subsequently, during the repeated hydration/dehydration cycles, measurements revealed that the Clariti‐20HEMA and Ultra‐20HEMA samples maintained remarkably consistent wettability throughout all five cycles. For the Acuvue‐20HEMA lenses, a slight increase in the contact angle was observed beginning at the fourth cycle. Nevertheless, the surface remained highly hydrophilic compared to the pristine Acuvue control. We attribute this slight variation to the Acuvue‐20HEMA variant possessing the thinnest coating among the investigated samples, rendering it relatively more susceptible to minor polymer desorption or delamination under severe cyclic osmotic stress (Figure 3j and Table 1). Finally, the samples underwent an accelerated aging test comprising three consecutive cycles of high‐temperature steam sterilization to concurrently evaluate the potential for polymer oxidation during this rigorous process. The resulting data verified that the robust hydrophilic layer on all three modified lenses was completely unaffected by extreme thermal conditions, where the characteristic poly(HEMA) signals (1,158 cm^−1^ (C─O─C) and 1,720 cm^−1^ (C═O)) in the ATR‐FTIR spectra remained prominent (Figure S12) [25]. Collectively, these comprehensive experimental findings provide compelling evidence supporting the excellent long‐term durability and clinical viability of the applied poly(HEMA) layer. In a distinct observation following the prolonged storage of HEMA modified contact lenses in bovine serum albumin and lysozyme solutions, the coating exhibited no enzymatic degradation. Because lysozyme, the most abundant enzyme in human tear fluid, specifically targets and cleaves *β*‐(1,4)‐glycosidic bonds [37], it does not induce enzymatic degradation of the poly(HEMA) network. Instead, the prolonged immersion led to protein deposition on the lens surface. These deposited proteins subsequently underwent surface rearrangements and conformational changes [38], which ultimately resulted in a lower water contact angle and further enhanced the overall surface wettability (Figure S11d) [39].

While certain macromolecules, particularly thermoresponsive polymers, experience hydration shell disruption and a depression of their lower critical solution temperature (LCST) in salt‐containing buffers [40], poly(HEMA) operates via a fundamentally different hydration mechanism. Unlike thermoresponsive materials, poly(HEMA) does not exhibit an LCST [41]. Instead, its robust hydrophilicity is governed by highly stable hydrogen‐bonding networks formed between its abundant hydroxyl groups and surrounding water molecules. Consequently, the hydration state of poly(HEMA) remains intact without salt‐induced structural collapse. This fundamental mechanistic stability elucidates our experimental observations, confirming that the coating performance of the modified lenses remains completely unaffected following prolonged immersion in physiological saline or simulated tear fluid at 35°C (Figure S11a–c).

To visualize this 3D molecular entanglement with higher spatial resolution, we performed ToF‐SIMS depth profiling. We selected Acuvue‐20HEMA as the representative model, as the Acuvue substrate exhibited the highest surface coverage among the tested lens types, while the 20% formulation yielded the maximal coating thickness within the Acuvue series. By utilizing the C_2_H_5_O^+^ fragment as a marker for poly(HEMA) and the SiC_3_H_9_^+^ fragment for the siloxane substrate, the reconstructed 3D images reveal a clear HEMA‐enriched layer localized at the near‐surface region of Acuvue‐20HEMA, in contrast to the pristine Acuvue control. (Figure 4a–d). Crucially, lateral chemical mapping of the Acuvue‐20HEMA demonstrates a striking transition: the outermost surface is dominated exclusively by poly(HEMA) signals (red), whereas the subsurface layer (20 s sputtering) reveals a distinct coexistence of poly(HEMA) and siloxane signals (Figure 4e,f). This molecular intermingling provides direct evidence of semi‐IPN formation, with hydrophilic HEMA chains physically penetrating and anchoring within the silicone hydrogel network. Moreover, analogous chemical transitions and semi‐IPN structure were consistently observed in the modified Clariti and Ultra substrates, confirming the broad universality of our surface engineering strategy (Figures S17 and S18).

**FIGURE 4:**
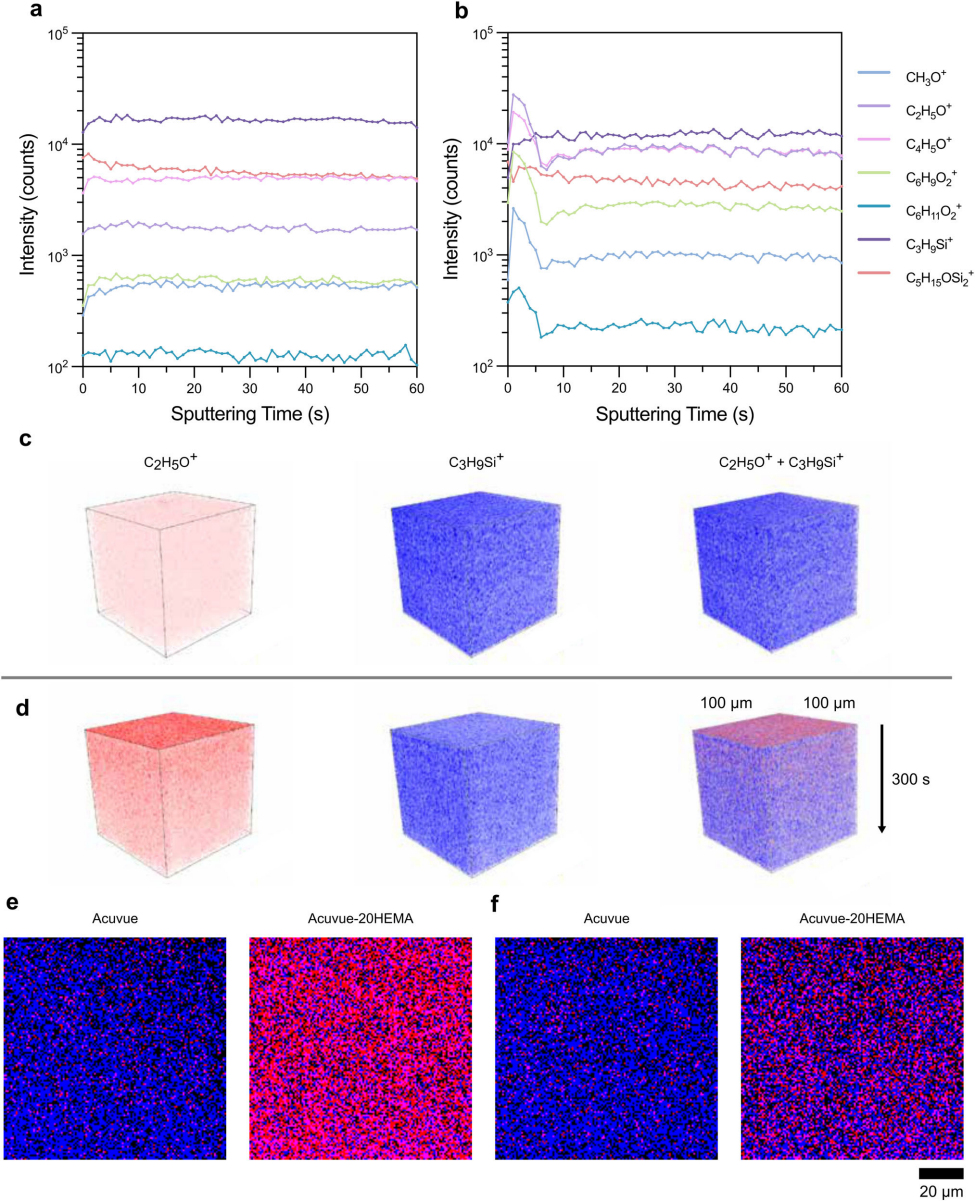
ToF‐SIMS characterization of HEMA‐modified contact lenses. (a,b) Depth profiles acquired using GCIB sputtering for pristine Acuvue (a) and Acuvue‐20HEMA (b) lenses. (c,d) Three‐dimensional reconstructed images (100 × 100 µm^2^) of pristine Acuvue (c) and Acuvue‐20HEMA (d) lenses. The panels display the spatial distribution of the HEMA‐specific fragment C_2_H_5_O^+^ (red), the siloxane‐specific fragment C_3_H_9_Si^+^ (blue), and their overlay. The vertical axis corresponds to a total sputtering time of 300 s. (e,f) Lateral overlap chemical maps comparing Acuvue and Acuvue‐20HEMA at surface (e) and a sputtering time of 20 s (f). Red and blue pixels represent C_2_H_5_O^+^ and C_3_H_9_Si^+^ signals, respectively. Scale bar, 20 µm.

Among all HEMA‐modified lenses, the 20% formulation generally yielded the highest surface coverage. A notable exception was observed in the Acuvue platform, where both Acuvue‐10HEMA and Acuvue‐20HEMA reached a saturation plateau, achieving maximal coverage that significantly exceeded that of Clariti‐20HEMA and Ultra‐20HEMA. Regarding vertical architecture, the 20% formulation consistently produced the thickest coating and semi‐IPN structures within each respective lens group. Interestingly, while semi‐IPN thickness appeared to be governed by monomer concentration, showing consistent values across different substrates, Clariti‐20HEMA exhibited the absolute thickest discrete coating layer. Nevertheless, upon comprehensive evaluation, Acuvue‐20HEMA was identified as the optimal representative model, as it combines the critical advantage of saturated surface coverage with a substantial coating thickness, which represents the maximal thickness within the superior Acuvue series.

### 2.2 Enhanced Surface Properties of HEMA‐Modified Contact Lenses

To translate structural engineering into functional performance, we evaluated the surface wettability and homogeneity of the modified interfaces. This hydrophilic poly(HEMA) layer effectively masks the inherent hydrophobic surface of the silicone hydrogel to yield the modified lens. All HEMA‐modified lenses exhibited significantly reduced water contact angles compared to pristine controls (Figure 5a). Notably, the static contact angle was found to be directly correlated with poly(HEMA) surface coverage across all HEMA‐modified lenses. Specifically, Acuvue‐10HEMA and Acuvue‐20HEMA exhibited comparable low contact angles (54.76° ± 1.12° and 55.64° ± 0.85°, respectively), a result attributed to their mutually saturated surface coverage (85.87% ± 5.10% and 88.92% ± 2.27%). In contrast, for the other lens substrates, the 20% formulation consistently displayed superior hydrophilicity compared to the 10% counterparts, driven by the significantly higher surface coverage achieved at the increased monomer concentration (Figure 1c and Table 1). In terms of dynamic contact angle, the receding angle remained remarkably consistent when comparing the pristine Acuvue to the modified Acuvue‐20HEMA (from 21.48° ± 0.72° to 22.16° ± 0.60°). However, the advancing angle exhibited a notable decrease from 38.57° ± 0.39° to 29.28° ± 3.35°. This reduction indicates that the originally abundant hydrophobic domains on the pristine Acuvue surface were successfully covered by the newly grafted poly(HEMA) network. Consequently, the contact angle hysteresis, defined as the difference between the advancing and receding angles, decreased significantly from 17.09° ± 0.82° for Acuvue to 7.12° ± 3.40° for Acuvue‐20HEMA (Figure S13). This minimized hysteresis clearly demonstrates that the original heterogeneous surface, characterized by micro‐phase separation between hydrophilic and hydrophobic regions, was successfully transformed into a highly uniform and continuous hydrophilic poly(HEMA) phase.

**FIGURE 5:**
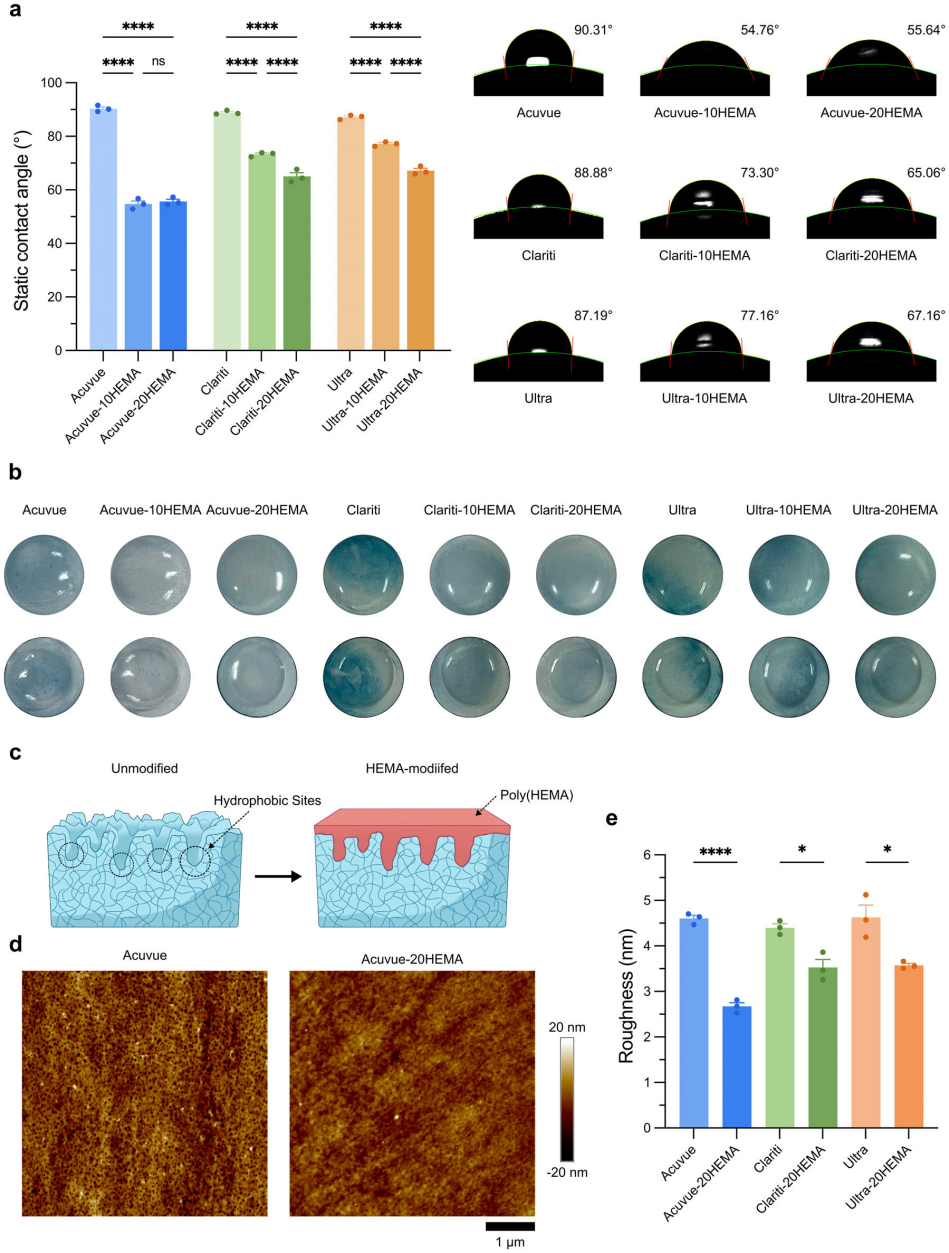
Improved surface properties of HEMA‐modified contact lenses. (a) Static water contact angle measurements for pristine and HEMA‐modified (10% and 20% HEMA) Acuvue (blue), Clariti (green), and Ultra (orange) lenses. Representative optical images of water droplets on the lens surfaces are shown on the right, indicating enhanced hydrophilicity after surface modification. (b) Macroscopic photographs of lenses stained with Solvent Black 3, a dye that selectively binds to hydrophobic domains. The panels display lenses in convex‐up (top row) and concave‐up (bottom row) orientations. (c) Schematic illustration demonstrating how the poly(HEMA) coating completely smooths the initially rough surface of the unmodified lens. (d) Representative AFM topography images of pristine Acuvue and Acuvue‐20HEMA surfaces. The scale bar represents 1 µm; the vertical color scale ranges from −20 nm to +20 nm. (e) Quantitative analysis of surface roughness (*R*_q_) derived from AFM measurements, showing a reduction in surface roughness upon modification. Data are presented as the mean ± s.e.m. (*n* = 3). Statistical significance was calculated using one‐way ANOVA: ns, not significant (*p* > 0.05), *p < 0.05, ****p < 0.0001. The schematic in panel (c) was prepared using Inkscape, with selected visual elements generated using Gemini Nano Banana 2 and subsequently edited and integrated in Inkscape. All scientific content was reviewed and verified by the authors.

Macroscopic homogeneity was visualized using Solvent Black 3, a lipophilic dye that targets hydrophobic siloxane domains [42]. While all pristine lenses displayed heterogeneous hydrophobic patches, modified surfaces, particularly Acuvue‐20HEMA, showed negligible dye retention, confirming the formation of a uniform hydrophilic layer (Figure 5b). The unmodified contact lens exhibits a rough surface due to differential swelling caused by hydrophilic‐hydrophobic micro‐phase separation. Following surface modification, the application of a poly(HEMA) layer completely covers the hydrophobic sites, resulting in a significantly smoother surface (Figure 5c). This homogenization was further corroborated by AFM topography (Figure 5d and Figure S19). Consequently, surface roughness (*R*_q_) decreased markedly by 42% for Acuvue‐20HEMA relative to the pristine Acuvue control, whereas Clariti‐20HEMA and Ultra‐20HEMA exhibited more moderate reductions of 20% and 23%, respectively. The superior smoothness of the Acuvue‐20HEMA bio‐interface is attributed to its highest poly(HEMA) surface coverage (Figure 5e and Table 1).

Ensuring biosafety is a prerequisite for clinical application. Cytotoxicity assays using L929 and V79 fibroblast cells confirmed that cell viability remained above the 70% threshold (ISO 10993–5) for all pristine and HEMA‐modified lenses, verifying that the washing steps effectively removed residual reagents (Figure S20).

Finally, we assessed the surface resistance to non‐specific biofouling. In pristine silicone hydrogels, the exposed hydrophobic siloxane domains act as preferential anchoring sites for proteins, creating a conditioning film that subsequently facilitates cell attachment [38]. Our results demonstrate that the anchored poly(HEMA) effectively masks these bio‐active regions. Consequently, the adsorption of both bovine serum albumin (BSA) and lysozyme was significantly suppressed (Figure S21). This inhibition of protein fouling directly translated to minimal L929 cell adhesion (Figure S22), validating the protective function of the hydrophilic interface against the protein‐cell adsorption cascade [43].

Across all three lens types, the 20% HEMA variants consistently demonstrated superior antifouling efficacy compared to their 10% counterparts. For Clariti and Ultra, this performance enhancement is primarily driven by the notably higher poly(HEMA) surface coverage achieved with the 20% formulation. Despite achieving equivalent surface coverage (Figure 1c), Acuvue‐20HEMA still exhibited superior antifouling performance compared to Acuvue‐10HEMA. This specific enhancement is attributed to the greater thickness of the poly(HEMA) layer and semi‐IPN structure (Figure 3j), underscoring that minimizing bio‐adhesion requires not only high surface coverage but also a sufficient physical barrier thickness (Table 1) [44].

### 2.3 Preservation of Intrinsic Lens Properties Following Surface Modification

Beyond surface enhancements, preserving the fundamental material characteristics of the contact lenses is paramount. We confirmed that the surface engineering process did not compromise optical transparency, oxygen permeability, mechanical properties, or equilibrium water content (EWC). Optical transmittance spectra remained virtually superimposable across the full visible and ultraviolet spectral ranges for all lens types following modification (Figure 6a; Figure S23). Pristine and HEMA‐modified Acuvue lenses retained compliance with UV Class I standards (UVA < 10% and UVB < 1%), while pristine and HEMA‐modified Clariti and Ultra lenses consistently met UV Class II requirements (UVA < 50% and UVB < 5%) (Table S3). These data indicate that the organic solvents and reagents used during reaction neither leached intrinsic UV blockers nor induced matrix clouding, regardless of the HEMA concentration. Preserving this inherent UV‐blocking capability is critical for clinical safety, ensuring that the surface modification does not impair the lens's ability to shield ocular tissues from the deleterious effects of ultraviolet radiation.

**FIGURE 6:**
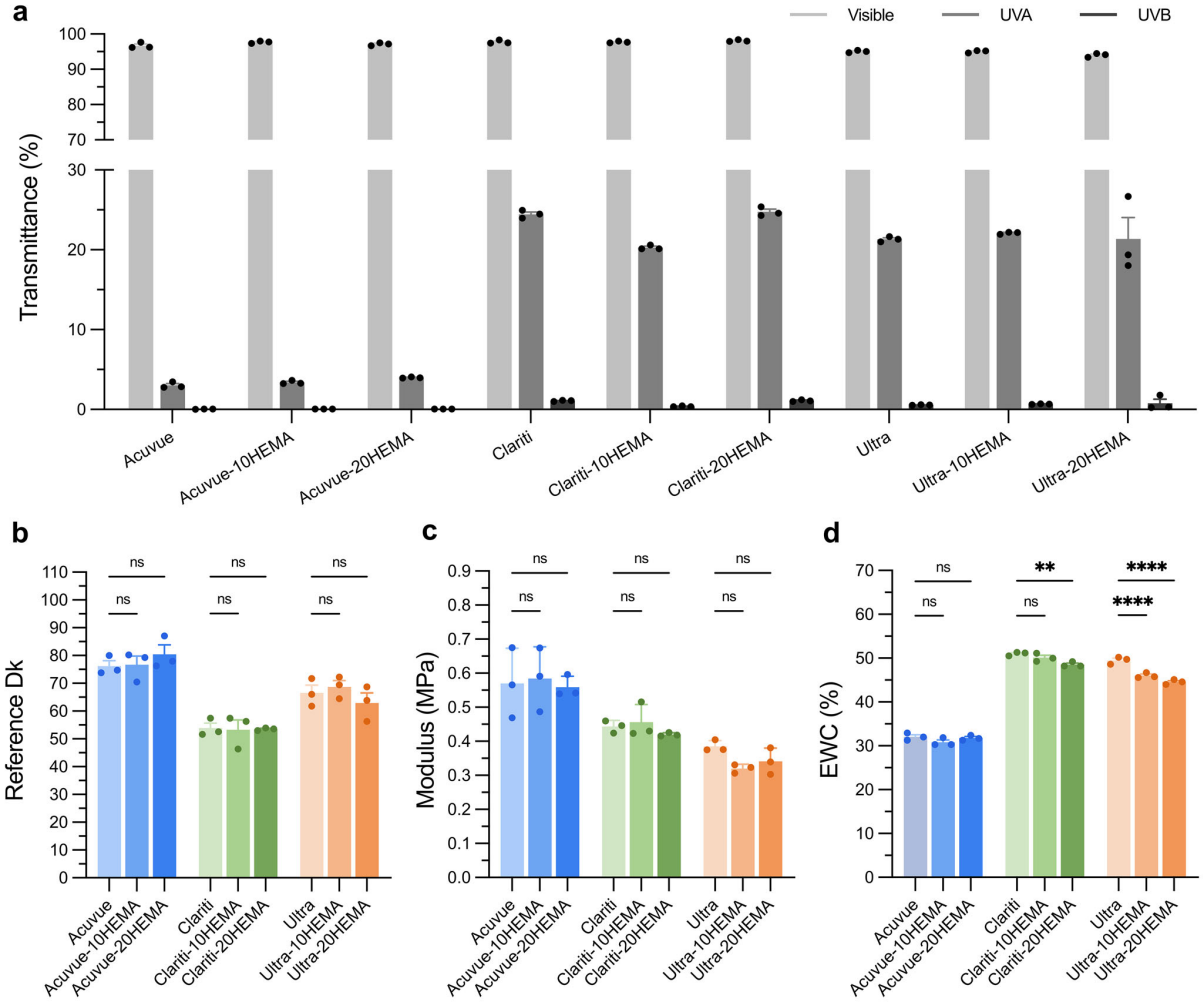
Optical and physicochemical characterization of HEMA‐modified contact lenses. (a) Light transmittance profiles in the visible (380–780 nm), UVA (316–380 nm), and UVB (280–315 nm) regions for pristine and HEMA‐modified (10% and 20% HEMA) Acuvue (blue), Clariti (green), and Ultra (orange) lenses. (b–d), Quantitative assessment of essential material properties: oxygen permeability (b), mechanical properties (c), and equilibrium water content (EWC) (d). Data are presented as mean ± s.e.m. (*n* = 3). Statistical significance was calculated using one‐way ANOVA: ns, not significant (*p* > 0.05), **p < 0.01, ****p < 0.0001.

Most importantly, the high oxygen permeability (Dk), a defining feature of silicone hydrogels, was fully maintained (Figure 6b). Calibrated against rigid gas‐permeable (RGP) reference standards (Figure S24), the measurements confirm that the poly(HEMA) layer and semi‐IPN structure (< 100 nm) present a negligible barrier to oxygen diffusion. Transport remains governed by the continuous siloxane channels within the bulk lens matrix.

Similarly, under fully hydrated physiological conditions, the bulk mechanical properties, including both tensile strength and macroscopic Young's modulus, remained statistically unchanged (Figure 6c and Figure S25), confirming that the modification is strictly confined to the interface without altering the underlying bulk polymer network. In stark contrast, the surface stiffness was significantly altered following the functionalization. As determined from the AFM force curves, the microscopic Young's modulus of the grafted coating is substantially lower than both the macroscopic tensile modulus and the microscopic surface modulus of the pristine lens obtained via QNM mode (Figure 6c and Table S2). This distinct gradient in stiffness is fundamentally attributed to the divergent polymeric architectures. Specifically, the surface‐grafted poly(HEMA) primarily behaves as a highly hydrated linear polymer, whereas the pristine contact lens comprises a densely crosslinked polymer network. Consequently, the modified lens features an exceptionally soft outermost layer, which is highly advantageous for reducing mechanical friction in physiological environments. Finally, EWC showed only a marginal decrease (∼5%) for Clariti‐20HEMA, Ultra‐10HEMA, and Ultra‐20HEMA (Figure 6d). This slight reduction is attributed to the significant decrease in surface roughness which lowers the effective specific surface area available for surface water retention.

Taken together, these extensive evaluations confirm that the proposed surface modification strategy successfully imparts highly desirable interfacial properties while strictly preserving the fundamental clinical requirements of the contact lenses. In particular, the engineering process did not compromise the optical transparency, essential UV blocking capabilities, high oxygen permeability, bulk mechanical properties, or equilibrium water content of the underlying substrates (Figure 6). Furthermore, the HEMA‐modified lenses demonstrated exceptional robustness when subjected to practical challenges mimicking actual clinical operations. The applied coating maintained its structural stability and chemical properties following rigorous simulated cleaning and disinfection protocols via repeated extreme steam sterilization. In complex biological environments, the modified surface not only exhibited superior resistance to initial protein adhesion but also benefited from prolonged protein immersion, which induced favorable surface conformational rearrangements that further enhanced overall wettability. Coupled with verified stability during extended storage in various solutions, these comprehensive findings strongly support the clinical viability and advanced biomedical application potential of the developed platform (Figure S11a–c,g).

### 2.4 Drug‐Functionalized Applications of HEMA‐Modified Contact Lenses

Among the evaluated substrates, the Acuvue platform was selected for drug functionalization as it exhibited the most effective surface modification. Specifically, both Acuvue‐10HEMA and Acuvue‐20HEMA demonstrated the superior grafting efficiency, achieving the highest poly(HEMA) surface coverage (∼89%) among all tested groups (Figure 1c). To enable on‐demand therapy, we synthesized MA‐NB‐DXMS, a multifunctional prodrug monomer integrating a polymerizable methacrylate group, a photocleavable *o*‐nitrobenzyl linker [45], and a dexamethasone moiety for anti‐inflammatory efficacy (Figure 7a). This design specifically addresses the clinical challenges of premature drug elution and thermal degradation during the steam sterilization process associated with conventional soaking methods.

**FIGURE 7:**
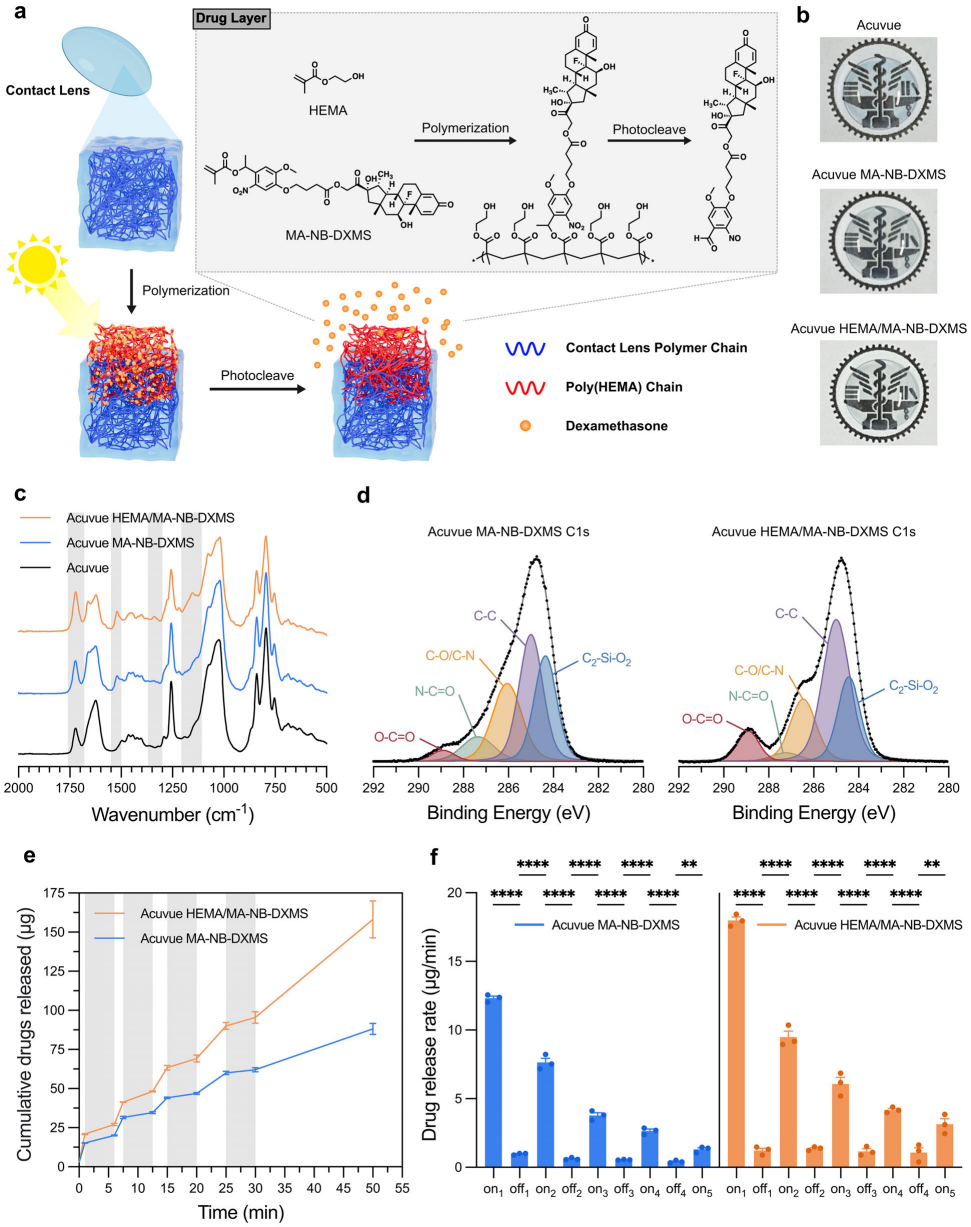
Fabrication and characterization of light‐responsive drug‐releasing contact lenses. (a) Schematic strategy for the surface immobilization of dexamethasone (DXMS). A photocleavable drug monomer (MA‐NB‐DXMS) is copolymerized with HEMA onto the lens surface, enabling light‐triggered drug release. (b) Optical photographs of pristine Acuvue, Acuvue MA‐NB‐DXMS, and Acuvue HEMA/MA‐NB‐DXMS lenses placed over an NYCU emblem to demonstrate optical transparency. (c) ATR‐FTIR spectra (500–2000 cm^−1^) of pristine Acuvue (black), Acuvue MA‐NB‐DXMS (blue), and Acuvue HEMA/MA‐NB‐DXMS (orange) lenses. (d) High‐resolution C1s XPS spectra of Acuvue MA‐NB‐DXMS and Acuvue HEMA/MA‐NB‐DXMS. (e) Cumulative DXMS release profiles under alternating cycles of light stimulation (white regions) and darkness (grey regions). (f) Corresponding drug release rates during the light‐on and light‐off intervals. Data are presented as mean ± s.e.m. (*n* = 3). Statistical significance was calculated using one‐way ANOVA: **p < 0.01, ****p < 0.0001. The schematic in panel (a) was prepared using Inkscape; 3D elements were modeled and rendered in Blender.

A prerequisite for clinical translation is thermal stability under standard sterilization conditions. HPLC analysis and high‐resolution mass spectrometry confirmed that the prodrug withstands steam sterilization (121°C) without structural degradation or hydrolysis, retaining retention times identical to the non‐sterilized control (Figure S27).

Following stability validation, we grafted either MA‐NB‐DXMS alone or a HEMA/MA‐NB‐DXMS copolymer onto the lens surface. ATR‐FTIR characterization confirmed successful surface functionalization. Prominent absorption bands were observed at 1,158 and 1,720 cm^−1^, corresponding to the C─O─C and C═O stretching vibrations of the methacrylate ester linkages, respectively [25]. The appearance of additional distinct bands at 1,300–1,370 cm^−1^ and 1,500–1550 cm^−1^, assigned to the symmetric and asymmetric stretching of the nitro (NO_2_) group, definitively verified the incorporation of the light‐responsive prodrug (Figure 7c). Surface composition was further resolved by XPS. In the C1s spectra of both Acuvue MA‐NB‐DXMS and Acuvue HEMA/MA‐NB‐DXMS, a characteristic ester signal (O─C═O, 288.5 eV) was observed. However, the intensity of this peak was notably weaker in Acuvue MA‐NB‐DXMS, as it originates solely from the ester group of the prodrug, whereas Acuvue HEMA/MA‐NB‐DXMS exhibits a significantly stronger signal due to the additive contribution from the poly(HEMA) side chains (Figure 7d). Furthermore, relative to the pristine Acuvue control, distinct fluorine signals (F 1s) specific to dexamethasone were detected in both modified groups, substantiating the successful immobilization of the prodrug in both formulations (Figure S28).

We subsequently evaluated the light‐triggered release profiles using 405 nm visible light. The drug release was strictly modulated by light exposure, initiating upon illumination and ceasing in the dark (Figure 7e,f and Figure S29c,d). Quantification was achieved using a standard calibration curve of the photocleaved product (Figure S29b). Interestingly, while the mono‐functionalized lenses (Acuvue MA‐NB‐DXMS) exhibited sharp on/off switching, the copolymerized system (Acuvue HEMA/MA‐NB‐DXMS) displayed a secondary, sustained low‐level release during dark intervals. We attribute this phenomenon to the physical entrapment of cleaved drug molecules within the hydrated poly(HEMA) mesh, where the formation of hydrogen bonds between the drug and the polymer chains likely retards their diffusion [46]. Furthermore, the inclusion of HEMA enhanced the overall drug loading capacity by facilitating higher polymerization rates.

Importantly, unlike many drug‐loading strategies that often compromise optical clarity, this functionalization had no adverse effect on transmittance. The drug‐loaded lenses remained visually indistinguishable from pristine controls and compliant with UV Class I standards (Figure 7b and Figure S30 and Table S3). Specifically, we optimized the reaction formulation to 0.1% MA‐NB‐DXMS and 0.1% HEMA to preserve this crucial optical transparency and prevent phase separation. Notably, elevating the MA‐NB‐DXMS concentration to 0.5% induced severe phase separation, which drastically reduced the optical transmittance to 38.59 ± 3.52%. (Figure S31). Collectively, these findings validate our strategy as a highly effective platform for ocular drug delivery. It successfully anchors drug molecules to create a smart, light‐responsive therapeutic material. Furthermore, the modified lenses strictly preserve the pristine optical transparency essential for uncompromised vision and exhibit remarkable thermal stability to withstand standard high‐temperature steam sterilization.

## 3 Conclusion

In summary, this work establishes an integrated engineering–characterization–function framework that enables quantitative understanding of buried nanocoatings on complex curved biointerfaces. Rather than focusing solely on surface modification, we demonstrate that rational biointerface engineering requires simultaneous control and quantitative characterization of coating chemistry, nanoscale architecture, and interfacial mechanics to reveal their direct relationships with biological function. By integrating GCIB‐assisted XPS, ToF‐SIMS, and a newly developed AFM modulus‐based computational algorithm, we establish a multimodal analytical platform capable of non‐destructively resolving the three‐dimensional chemical, structural, and mechanical organization of buried multilayer nanocoatings with nanometer‐scale precision. This framework provides, for the first time, quantitative visualization of vertical chemical gradients, discrete layer thicknesses, and interfacial mechanical distributions within soft, curved hydrogel substrates, overcoming a longstanding limitation in the characterization of conformal biomedical coatings. The quantitative insights obtained from this platform reveal how nanoscale coating architecture governs interfacial performance. The engineered semi‐interpenetrating polymer network (semi‐IPN) forms a robust buried interface that achieves nearly complete masking of hydrophobic silicone domains while preserving the intrinsic oxygen‐transport pathways, thereby simultaneously enhancing wettability, suppressing protein adsorption and cellular fouling, and maintaining optical transparency, UV protection, mechanical integrity, and oxygen permeability. Furthermore, the platform readily accommodates functional monomers, enabling homogeneous incorporation of a light‐responsive prodrug for predictable and sustained therapeutic release without compromising device performance. Beyond silicone hydrogel contact lenses, this work establishes a generalizable strategy for quantitatively engineering and characterizing multifunctional nanocoatings across diverse curved biomedical interfaces. More broadly, by linking coating chemistry, nanoscale architecture, interfacial mechanics, and biological function within a unified quantitative framework, this study provides a foundation for predictive biointerface engineering and offers a general paradigm for the rational design of next‐generation therapeutic coatings, implantable devices, and advanced biomedical interfaces.

## 4 Experimental Section

### 4.1 Preparation of Surface‐Modified poly(HEMA) Contact Lenses

The initiator solution was prepared by dissolving VA 086 (50 mg) in distilled water (5 mL). The 20% monomer solution was prepared by mixing HEMA (1 mL) with 50% isopropanol (IPA, 4 mL). Prior to use, both solutions were purged with nitrogen gas for 30 min to eliminate dissolved oxygen. Pre‐dried contact lenses were immersed in the initiator solution for 30 min to allow initiator adsorption. The initiator‐laden lenses were then transferred to the monomer solution and subjected to thermally initiated free radical polymerization at 80°C for 30 min. After polymerization, the modified lenses were thoroughly washed with 75% ethanol to remove unreacted HEMA monomer and oligomers. The HEMA‐modified lenses were stored in PBS at room temperature until further characterization. This same procedure was applied to prepare lenses modified with 2.5% and 10% HEMA solutions.

### 4.2 Preparation of Surface‐Modified Light‐Responsive Dexamethasone‐Releasing Contact Lenses

The preparation procedure was similar to that described previously, except that the monomer solution consisted of either 0.1 % MA‐NB‐DXMS alone or a mixture of 0.1 % MA‐NB‐DXMS and 0.1 % HEMA, both dissolved in 50% IPA. The solution was then subjected to thermal free‐radical polymerization in an autoclave at 121°C. After the reaction, the lenses were allowed to cool to room temperature and subsequently rinsed in 75% ethanol to remove any unreacted monomers and oligomers. Finally, the surface‐modified light‐responsive dexamethasone‐releasing contact lenses were stored in PBS at room temperature until further use.

### 4.3 X‐Ray Photoelectron Spectroscopy

For surface characterization, the dried samples were first mounted onto the sample holder. The surface elemental composition and chemical states were analyzed by XPS using a PHI 5000 VersaProbe III system (ULVAC‐PHI Inc., Japan) equipped with a monochromatic Al Kα x‐ray source (h𝑣 = 1486.6 eV). To prevent surface charging on the insulating polymer samples, a dual‐beam charge neutralization system was employed during the measurements. Survey scans were acquired over a binding energy range of 0–1350 eV with a pass energy of 280 eV for qualitative elemental analysis. For detailed quantitative analysis, high‐resolution spectra of the C1s, O1s, N1s, and Si2p core‐level peaks were obtained with a pass energy of 55 eV. Depth profiling was conducted by incrementally sputtering the surface with a 10 kV Argon gas cluster ion beam (GCIB) for 5‐minute intervals over six cycles. All collected spectra were processed and analyzed using MultiPak and CasaXPS software packages. The binding energy scale was strictly calibrated by setting the C‐C component of the C1s peak to 285.0 eV.

The surface coverage of the poly(HEMA) layer on the HEMA‐modified lens was quantified from the XPS high‐resolution spectra. The coverage was defined as the ratio of the integral area of the ester bond signal originating exclusively from the poly(HEMA) layer ($A_{\mathrm{modified},O-C\;=\;O}^{\mathrm{pHEMA}}$) to the total C1s integral area on the HEMA‐modified lens (*A*_modified,O − C  =  O_), as shown in Equation (1):

(1) $$\mathrm{Cove}\mathrm{rage}\;\left(\%\right)=\frac{A_{\mathrm{modified},O-C=O}^{\mathrm{pHEMA}}}{A_{\mathrm{modified},O-C=O}}\times 100\%$$

To isolate the ester bond signal from poly(HEMA) ($A_{\mathrm{modified},O-C\;=\;O}^{\mathrm{pHEMA}}$), the contribution from the underlying base lens was subtracted from the total O─C═O signal measured on the HEMA‐modified lens (*A*_modified,O − C  =  O_). The Si2p signal, which is unique to the silicone hydrogel base lens, was used as an internal reference to correct for signal attenuation. The corrected ester bond area was calculated using Equation (2):

(2) $$A_{\mathrm{modified},O-C\;=\;O}^{\mathrm{pHEMA}}=A_{\mathrm{modified},O-C=O}\;-\left(A_{\mathrm{base},O-C\;=\;O}\times \frac{A_{\mathrm{modified},\mathrm{Si}2p}}{A_{\mathrm{base},\mathrm{Si}2p}}\right)$$

where *A*_base,O − C  =  O_ and *A*_base,Si2p_ are the integral areas of the O─C═O and Si2p signals from the pristine lens, respectively, while *A*_modified,Si2p_ is the Si2p signal area from the HEMA‐modified lens.

### 4.4 Atomic Force Microscope (AFM)

AFM measurements, including surface topography for roughness analysis and force‐distance curve (FDC) acquisition, were conducted in an aqueous environment using a Dimension Icon AFM system (Bruker Corp., USA) equipped with a liquid cell. This method is a modification of a previously reported technique [24]. For all measurements, rectangular‐shaped, gold‐coated silicon nitride cantilevers (NITRA‐TALL‐R‐G, AppNano, Ltd., USA) featuring a silicon tip and a nominal spring constant of 0.1 N m^−1^ were employed.

For surface roughness measurement, topography images were acquired over 5 × 5 µm^2^ areas in the “PeakForce QNM in fluid” mode. The root mean square roughness (*R*_q_) was calculated from these topography images using NanoScope Analysis software.

To resolve the nanomechanical architecture, FDCs were collected within 1 × 1 µm^2^ regions using the “Point and Shoot” function. Each FDC was generated with approach and retract velocities of 800 nm s^−1^ and an applied force in the range of 8—12 nN. Nanomechanical properties were extracted from FDCs using two self‐consistent algorithms tailored for the unmodified and the surface‐modified lens. Both of the algorithms were implemented in Python 3.6. The analysis is based on the Hertz model for a spherical indenter. The applied force (*F*) on the sample was calculated from the cantilever deflection (*d*) using Hooke's law, *F* = *kd*, where *k* is the cantilever's nominal spring constant. The indentation depth (*δ*) was calculated as the difference between the piezo displacement (*z*) and the cantilever deflection (*δ* = *z*—*d*). The analysis assumes the Hertz model for a spherical indenter, where the relationship between force and indentation depth is given by:

(3) $$F=\frac{4}{3}\;E^{\prime }R^{\frac{1}{2}}\delta^{\frac{3}{2}}$$

where *R* is the tip radius. The reduced modulus(*E'*), is related to the sample's Young's modulus (*E*) and Poisson's ratio (*ν)* by:

(4) $$E^{\prime }=\frac{E}{1-\nu^{2}}\;$$

where *ν* is defaulted to 0.5.

For unmodified contact lenses, a self‐consistent modulus algorithm was implemented to precisely identify the contact point (Figure 2b). Briefly, an initial contact point was estimated using a deflection threshold (1.5*σ* above baseline noise). The deflection‐displacement data were converted to experimental *F*‐*δ* curves, and the reduced modulus was derived from the slope of the linearized *δ* ^1.5^‐*F* plot according to Equation (3). The true contact point was subsequently refined by minimizing the mean squared error (MSE) between the experimental data and the theoretical Hertzian fit.

For surface‐modified contact lenses possessing a multi‐layered structure, an advanced iterative algorithm was employed to simultaneously decouple the mechanical properties of the coating (*E'*_coating_) and base lens (*E'*_base_), and to quantify layer thicknesses (*t*_coating_ and *t*_semi‐IPN_) (Figure 2b). The procedure involves a global optimization loop that identifies both the surface contact point and the coating/semi‐IPN interface. Convergence was defined when the MSE between the experimental and theoretical Hertzian curves for the coating layer was minimized, and the discrepancy between moduli derived from curve fitting vs. linear regression remained within a pre‐defined tolerance. Following the determination of the coating modulus, *E'*_base_ was extracted from linear regression of the force curve in the deep indentation regime. The lower boundary of the semi‐IPN structure was defined using a 5*σ* deflection threshold relative to the base lens signal. Finally, discrete layer thicknesses were calculated based on the indentation depth corresponding to these identified structural transitions.

### 4.5 Drug Release Test

Qualitative and quantitative analysis of drug concentrations in the samples was performed using a Waters 1525 Binary HPLC Pump (Waters Corp., USA) equipped with a Waters 2489 UV/Visible Detector. Prior to injection, all samples were filtered through a 0.22 µm PTFE syringe filter. Quantification was conducted using the external standard calibration method, in which calibration curves were constructed by plotting the peak area against known concentrations of the drug standard.

The chromatographic separation was achieved using a C18 reversed‐phase column (19 mm × 150 mm, 10 µm, XTERRA Prep RP 18 OBD) with a mobile phase consisting of acetonitrile and deionized water. The mobile phase was filtered through a 0.22 µm membrane and degassed before use. The analysis was carried out at a flow rate of 8 mL/min using gradient elution with 10% acetonitrile and 90% deionized water. Detection was performed at a wavelength of 350 nm, with an injection volume of 200 µL at ambient temperature. The run time for each analysis was 20 min, adjusted as needed according to the retention time of the analytes.

For the drug release test, the modified contact lens was immersed in a 2 mL acetonitrile sample vial and protected from light. At specified time intervals, 1.5 mL of the solution was withdrawn and stored at low temperature for subsequent analysis, and the withdrawn volume was immediately replaced with an equal volume of fresh acetonitrile. The sample was also alternately irradiated with 405 nm ultraviolet light at an intensity of 10 mW/cm^2^ for defined periods, and samples were collected after cumulative irradiation times of 1, 2.5, 5, 10, and 30 min.

To construct the calibration curve for the photolysis product, a 1 mM stock solution of MA‐NB‐DXMS in acetonitrile was first irradiated at 365 nm (10 mW/cm^2^) for 30 min. The irradiated solution was then serially diluted to obtain standard concentrations of 1.0, 0.5, 0.1, 0.05, 0.01, 0.005, and 0.001 mM. These standard solutions were subsequently analyzed by HPLC, and the photolysis product was quantified based on its integrated peak area at a retention time of approximately 11–12 min.

### 4.6 Supplementary Methods

Detailed experimental protocols regarding chemical synthesis and structural characterization, including ATR‐FTIR, ToF‐SIMS, NMR, and mass spectrometry, are provided in the Supporting Information. Furthermore, methodologies for material property evaluation, comprising static contact angle, Solvent Black 3 staining, optical properties, equilibrium water content (EWC), swelling ratio, mechanical properties, oxygen permeability, and accelerated aging, are described therein. Finally, protocols for biological assessments, such as cytotoxicity and adhesion assays for cells, BSA, and lysozyme, are also included.

### 4.7 Statistical Analysis

The data for all bar graphs were presented as mean ± s.e.m. Statistical analyses were performed using GraphPad Prism 10 (GraphPad Software). Comparisons between two groups were analyzed using two‐tailed Student's *t*‐tests, whereas multiple group comparisons were performed using one‐way analysis of variance (ANOVA). Statistical significance is indicated as follows: ns, not significant (*p* > 0.05), *p < 0.05, **p < 0.01, ***p < 0.001, and ****p < 0.0001.

## Author Contributions

**Po‐Hsiang Chen**: methodology, investigation, data curation, validation, formal analysis, writing – original draft. **Jia‐Hua Wang**: investigation, data curation. **Ching Chen**: data curation. **Shih‐Hsuan Fu**: data curation. **Rajan Deepan Chakravarthy**: investigation. **Hsin‐Chieh Lin**: conceptualization, supervision, funding acquisition, project administration, resources, writing – review and editing.

## Conflicts of Interest

The authors declare no conflicts of interest.

## Supporting information




**Supporting File**: advs78148‐sup‐0001‐SuppMat.docx.

## Data Availability

The data that support the findings of this study are available within the article and its Supporting Information.
