# Supplementary material for: Nanoscale Buried Interface Control in Silicone Hydrogels Enables a Biointerface Engineering Platform
Source: Adv Sci (Weinh). 2026 Oct 19:e78148. Online ahead of print. doi: 10.1002/advs.78148 (PMC13679924; doi:10.1002/advs.78148)
Supplement: Supplementary file 1 [file ADVS-9999-e78148-s001.docx]

Supporting Information

**Nanoscale Buried Interface Control in Silicone Hydrogels Enables a Biointerface Engineering Platform**

*Po-Hsiang Chen, Jia-Hua Wang, Ching Chen, Shih-Hsuan Fu, Rajan Deepan Chakravarthy, and Hsin-Chieh Lin**

P. H. Chen, J. H. Wang, C. Chen, S. H. Fu, R. D. Chakravarthy

Department of Materials Science and Engineering, National Yang Ming Chiao Tung University, Hsinchu 300093, Taiwan, Republic of China

H. C. Lin

Department of Materials Science and Engineering, National Yang Ming Chiao Tung University, Hsinchu 300093, Taiwan, Republic of China

Center for Intelligent Drug Systems and Smart Bio-Devices (IDS^2^B), National Yang Ming Chiao Tung University, Hsinchu 30068, Taiwan, Republic of China

E-mail: hclin45@nycu.edu.tw

**CONTENTS**

**Supplementary Note 1.** Poly(HEMA) coverage and thickness analysis.	1

**Supplementary Note 2.** Estimation of Hydrazide-Functionalized Hyaluronic Acid (HA-Hzd) coverage	2

**Supplementary Note 3.** Estimation of Thiolated Hyaluronic Acid (HA-SH) coverage	3

**Supplementary Note 4.** Estimation of Poly(2-methacryloyloxyethyl phosphorylcholine) (PMPC) coverage	4

# Supplementary Materials and Methods……………………………………………………..5

# Chemical Synthesis…………………………………………………………………………..11

[**Table S1.** Atomic concentrations of the pristine and HEMA-modified contact lenses determined via XPS.	18](#_Toc238127842)

[**Table S2.** Quantitative nanomechanical properties and layer thicknesses derived from AFM force–distance analysis	18](#_Toc238127843)

[**Table S3.** Optical transmittance and UV-blocking classification of pristine and surface-modified lenses	19](#_Toc238127844)

[**Figure S1.** ATR-FTIR spectra of pure poly(HEMA) compared with pristine and HEMA-modified lenses	20](#_Toc238127845)

[**Figure S2.** XPS survey spectra of pristine and HEMA-modified lenses	20](#_Toc238127846)

[**Figure S3.** Atomic concentrations of the pristine and HEMA-modified contact lenses determined via XPS.	21](#_Toc238127847)

[**Figure S4.** High-resolution XPS C1s spectra of pristine and HEMA-modified lenses	22](#_Toc238127848)

[**Figure S5.** High-resolution XPS O1s spectra of Acuvue and Acuvue-20HEMA	23](#_Toc238127849)

[**Figure S6.** Evolution of high-resolution C1s spectra of HEMA-modified lenses	23](#_Toc238127850)

[**Figure S7.** Depth-dependent poly(HEMA) coverage profiles during GCIB sputtering	23](#_Toc238127851)

[**Figure S8.** Representative cantilever deflection curves and linearized force–indentation plots for HEMA-modified Acuvue lenses	24](#_Toc238127852)

[**Figure S9.** Representative cantilever deflection curves and linearized force–indentation plots for pristine and HEMA-modified Clariti lenses	25](#_Toc238127853)

[**Figure S10.** Representative cantilever deflection curves and linearized force–indentation plots for pristine and HEMA-modified Ultra lenses	27](#_Toc238127854)

[**Figure S11.** Long-term stability and durability of HEMA-modified contact lenses	30](#_Toc238127855)

[**Figure S12.** Stability assessment via accelerated aging of HEMA-modified lenses	31](#_Toc238127856)

[**Figure S13.** Dynamic contact angles of pristine and HEMA-modified Acuvue contact lenses	32](#_Toc238127857)

[**Figure S14-S16.** Characteristic ToF-SIMS mass spectra of pristine and HEMA-modified lens surfaces	32](#_Toc238127858)

[**Figure S17-S18.** ToF-SIMS characterization of HEMA-modified contact lenses	35](#_Toc238127861)

[**Figure S19.** Representative AFM topography images comparing pristine and HEMA-modified lenses	39](#_Toc238127863)

[**Figure S20.** In vitro biocompatibility assessment of pristine and HEMA-modified lenses	40](#_Toc238127864)

[**Figure S21.** Protein antifouling performance of pristine and HEMA-modified contact lenses	40](#_Toc238127865)

[**Figure S22.** Cell antifouling performance of HEMA-modified contact lenses	41](#_Toc238127866)

[**Figure S23.** Optical transmission spectra of pristine and HEMA-modified contact lenses	41](#_Toc238127867)

[**Figure S24.** Calibration of oxygen permeability measurements using reference RGP lenses	42](#_Toc238127868)

[**Figure S25.** Tensile strength of pristine and HEMA-modified contact lenses	42](#_Toc238127869)

[**Figure S26.** Determination of intrinsic oxygen permeability (Dk) via the multi-lens stacking method	43](#_Toc238127870)

[**Figure S27.** Thermal stability and structural integrity of the photolabile prodrug	44](#_Toc238127871)

[**Figure S28.** XPS characterization of drug-loaded contact lenses	45](#_Toc238127872)

[**Figure S29.** HPLC verification of photocleavage kinetics and calibration for drug quantification	46](#_Toc238127873)

[**Figure S30.** Optical transmittance spectra comparing the pristine lens with the drug-loaded lens	47](#_Toc238127874)

[**Figure S31.** Optical transmittance spectra comparing the pristine Acuvue lens and the HEMA-modified drug-loaded lens prepared with a high-concentration formulation	47](#_Toc238127875)

[**Figure S32.** ¹H NMR spectrum of compound **1** in chloroform-*d*.	48](#_Toc238127876)

[**Figure S33.** ¹H NMR spectrum of compound **2** in chloroform-*d*.	48](#_Toc238127877)

[**Figure S34.** ¹H NMR spectrum of compound **3** in DMSO-*d*_6_.	49](#_Toc238127878)

[**Figure S35.** ¹H NMR spectrum of compound **4** in DMSO-*d*_6_.	49](#_Toc238127879)

[**Figure S36.** ¹H NMR spectrum of compound **5** in DMSO-*d*_6_.	50](#_Toc238127880)

[**Figure S37.** ¹H NMR spectrum of MA-NB-DXMS in DMSO-*d*_6_.	50](#_Toc238127881)

[**Figure S38.** HR-ESI-MS spectrum of MA-NB-DXMS.	51](#_Toc238127882)

**Reference**	52

**Supplementary Note 1****. Poly(HEMA) coverage and thickness analysis.**

The coverage difference is primarily attributed to variations in the swelling ratio among the lens materials. The swelling ratios for Acuvue, Clariti, and Ultra lenses were 47.2 ± 1.0%, 104.4 ± 1.1%, and 98.2 ± 1.9%, respectively, indicating that the Acuvue lens absorbed the lowest concentration of initiator during the surface modification process. According to the kinetics of radical polymerization, the kinetic chain length (𝜈) is proportional to the monomer concentration ([M]) and inversely proportional to the square root of the initiator concentration ([I]) , as described by the following relation:

$\nu\propto\left[ M \right]\left[ I \right]^{-\frac{1}{2}}$	(S1)

Consequently, at a fixed monomer concentration, the Acuvue HEMA-modified lens surface exhibited the longest poly(HEMA) chain length, resulting in the highest poly(HEMA) coverage.

Among these lenses, the HEMA-modified Clariti lenses developed the thickest poly(HEMA) layer. This outcome is primarily attributed to their swelling ratio of 104%, the highest among the three, suggesting a maximum absorption of the initiator during the modification process. According to the kinetics of radical polymerization, the rate of propagation (*R_p_*) is proportional to the monomer concentration ([M]) and the square root of the initiator concentration ([I]), as described by the following relation.

$R_{p} \propto\left[ M \right]\left[ I \right]^{\frac{1}{2}}$	(S2)

Consequently, the Clariti HEMA-modified lens exhibited the fastest propagation rate, resulting in a greater pHEMA thickness than the other two lenses at equivalent HEMA concentrations.

**Supplementary Note 2. Estimation of Hydrazide-Functionalized Hyaluronic Acid (HA-Hzd) coverage.**^[1]^

To estimate the surface coverage of the hydrazide-functionalized hyaluronic acid (HA-Hzd) coating on the oxidized pHEMA (Ox-pHEMA) substrate, the experimental nitrogen (N) atomic percentage obtained from XPS was compared to the theoretical nitrogen content of the pure HA-Hzd polymer.

Since XPS does not detect hydrogen, only non-hydrogen atoms are considered. The native HA disaccharide repeating unit (C_14_H_21_NO_11_) consists of 26 XPS-detectable atoms (14 C, 11 O, and 1 N). The modification with adipic acid dihydrazide (ADH, C_6_H_14_N_4_O_2_) involves the formation of an amide bond, releasing one water molecule (H_2_O) and adding a net C_6_N_4_O group. Consequently, the ADH-modified repeating unit consists of 37 XPS-detectable atoms (20 C, 12 O, and 5 N).

Based on the 53% degree of substitution determined experimentally, the average theoretical composition of the HA-Hzd polymer can be calculated as follows:

$$Theoretical N (\%)= \frac{\left( 47\%\times1 \right)+(53\%\times5)}{\left( 47\%\times26 \right)+(53\%\times37)}=9.80\%$$

XPS analysis revealed that the native and oxidized pHEMA hydrogels contained no detectable nitrogen, confirming that the N1s signal originates exclusively from the grafted HA-Hzd layer. Following modification, the HA-pHEMA sample exhibited a nitrogen content of approximately 1.0 atom%. The relative surface coverage of the HA-Hzd layer can be estimated by calculating the ratio of the experimentally measured nitrogen content to the theoretical limit of the pure polymer:

$$Coverage (\%)= \frac{Measured N (\%)}{Theorectical N (\%)}=\frac{1.00\%}{9.80\%}=10.2\%$$

This calculation indicates that approximately 10.2% of the atomic signal within the XPS sampling volume originates from the HA-Hzd coating, with the remaining signal primarily contributed by the underlying pHEMA substrate.

**Supplementary Note 3. Estimation of Thiolated Hyaluronic Acid (HA-SH) coverage.**^[2]^

To estimate the surface coverage of the thiolated hyaluronic acid (HA-SH) coating on the model silicone hydrogel (pHEMA-co-TRIS) substrate, the experimental nitrogen (N) atomic percentage obtained from angle-resolved XPS (AR-XPS) was compared to the theoretical nitrogen content of the pure HA-SH polymer.

Since XPS does not detect hydrogen, only non-hydrogen atoms are considered. The native HA disaccharide repeating unit (C_14_H_21_NO_11_) consists of 26 XPS-detectable atoms (14 C, 11 O, and 1 N). The modification with cysteamine replaces a hydroxyl group (-OH) with a cysteamine moiety (-NH-CH_2_-CH_2_-SH), adding a net C_2_NSO_-1_ group. Consequently, the thiolated repeating unit consists of 29 XPS-detectable atoms (16 C, 10 O, 2 N, and 1 S).

Based on the 32% degree of thiolation determined experimentally, the average theoretical composition of the HA-SH polymer can be calculated as follows:

$$Theoretical N (\%)= \frac{\left( 68\%\times1 \right)+\left( 32\%\times2 \right)}{\left( 68\%\times26 \right)+\left( 32\%\times29 \right)}=4.90\%$$

XPS analysis revealed that the pristine and acrylated pHEMA-co-TRIS hydrogels contained virtually no nitrogen, confirming that the N1s signal originates exclusively from the grafted HA-SH layer . Following modification, the HA-PHEMA-co-TRIS samples exhibited an average nitrogen content of 1.6 atom% across the three take-off angles (1.7% at 30°, 2.0% at 45°, and 1.1% 90°). The relative surface coverage of the HA-SH layer can be estimated by calculating the ratio of the average experimentally measured nitrogen content to the theoretical limit of the pure polymer:

$$Coverage (\%)= \frac{Measured N \left( \% \right)}{Theorectical N \left( \% \right)}=\frac{1.60\%}{4.90\%}=32.7\%$$

This calculation indicates that an average of approximately 32.7% of the atomic signal within the XPS sampling volume originates from the HA-SH coating, with the remaining signal primarily contributed by the underlying silicone hydrogel substrate.

**Supplementary Note 4. Estimation of Poly(2-methacryloyloxyethyl phosphorylcholine) (PMPC) coverage.**^[3]^

To estimate the surface coverage of the poly(2-methacryloyloxyethyl phosphorylcholine) (PMPC) coating on the contact lens (CL) substrate, the experimental phosphorus (P) atomic percentage obtained from XPS was compared to the theoretical phosphorus content of the pure PMPC polymer.

Since XPS does not detect hydrogen, only non-hydrogen atoms are considered. The 2-methacryloyloxyethyl phosphorylcholine (MPC) repeating unit (C_11_H_22_NO_6_P) consists of 19 XPS-detectable atoms (11 C, 6 O, 1 N, and 1 P). The theoretical phosphorus composition of the pure PMPC polymer can be calculated as follows:

$$Theoretical P (\%)= \frac{1}{19}=5.26\%$$

XPS analysis revealed that the pristine contact lenses contained no detectable phosphorus, confirming that the P2p signal originates exclusively from the grafted PMPC layer. Following modification, the CL-P (5:30) sample exhibited a phosphorus content of 2.33 atom%. The relative surface coverage of the PMPC layer can be estimated by calculating the ratio of the experimentally measured phosphorus content to the theoretical limit of the pure polymer:

$$Coverage \left( \% \right)= \frac{Measured P \left( \% \right)}{Theorectical P \left( \% \right)}= \frac{2.33\%}{5.26\%}=44.3\%$$

This calculation indicates that approximately 44.3% of the atomic signal within the XPS sampling volume originates from the PMPC coating, with the remaining signal primarily contributed by the underlying contact lens substrate.

# Supplementary Materials

All chemicals were used as received. The intermediate compounds (**1**–**5**) used for the synthesis of MA-NB-DXMS were prepared according to previously reported procedures with slight modifications^[4]^. Three different commercial silicone hydrogel contact lenses were selected for surface modification: Acuvue^®^ Oasys (Senofilcon A, from Johnson & Johnson Vision Care Inc.), Clariti^®^ 1 day (Somofilcon A, from CooperVision Manufacturing Ltd.), Ultra^®^ One Day (Kalifilcon A, from Bausch + Lomb Inc.).

# Supplementary Methods

**Attenuated total reflectance Fourier transform infrared spectroscopy (ATR-FTIR)**

The functional groups of the lenses were characterized using a FTIR spectrometer (Spectrum 100, PerkinElmer, Inc., USA) equipped with a Quest ATR diamond extended range accessory (Specac, Ltd., UK). For analysis, a dried lens was placed in direct contact with the ATR crystal. The absorbance spectra were recorded over a wavenumber range of 400–4,000 cm^-1^ range.

**Time-of-flight secondary ion mass spectrometry (ToF-SIMS)**

Prior to the measurements, the dried samples were mounted onto the sample holder. ToF-SIMS measurements were performed using a TOF-SIMS V spectrometer (IONTOF GmbH, Germany). Mass surface spectra were acquired using a pulsed 15 keV Bi_3_^+^ primary ion beam at a pulsed current of 0.3 pA. The analysis was conducted over a 100 × 100 µm^2^ area. For depth profiling, a 5 keV Ar_n_^+^ GCIB was employed as the sputtering beam. Charge compensation was achieved using a low-energy electron flood gun. Data acquisition and processing were performed using the SurfaceLab.

**Static contact angle (SCA)**

The surface wettability of the lenses was evaluated by measuring the SCA using a contact angle goniometer (Phoenix-MT, Surface Electro Optics, Ltd., Korea) via the sessile drop method. Prior to measurement, each lens was fully hydrated in PBS. Excess surface moisture was then gently blotted with Kimwipes, and the prepared lens was immediately mounted on a polypropylene holder to provide a stable measurement surface. A single 5 µL droplet of deionized water was deposited onto the center of the lens surface. A high-resolution digital image of the droplet was captured immediately upon deposition. The static contact angle was then calculated from the droplet profile in the captured image.

**Solvent black 3 staining**

The Solvent Black 3 staining protocol was adapted from a previously reported method, with minor modifications^[5]^, and was used to evaluate the macroscopic homogeneity of the lens surface. To prepare the staining solution, 2 g of Solvent Black 3 were dissolved in 100 g of silicone oil and stirred for 30 minutes. The mixture was then centrifuged at 12,000 rpm for 30 minutes, and the supernatant was collected and diluted with silicone oil to a final volume of 150 mL. The staining solution was stored at room temperature until use. For surface staining, each lens was first soaked in PBS overnight to ensure complete hydration. Excess PBS was carefully removed from the lens surface using Kimwipes. The hydrated lens was then immersed in a vial containing 5 mL of the Solvent Black 3 solution for 30 minutes. After staining, the lens was thoroughly rinsed several times with fresh PBS to remove any residual dye. Finally, after gently blotting off surface PBS, the stained lens was imaged to assess surface homogeneity.

**Optical transmittance**

The optical transmittance of the lenses was measured using a UV-Vis spectrophotometer (V670, Jasco, Inc., Japan), with the procedure adhering to the ISO 18369-3:2017. For each measurement, a fully hydrated lens was placed in a quartz cuvette filled with PBS. A baseline correction was performed prior to sample analysis using a reference cuvette containing only PBS. The transmittance spectra were recorded across a wavelength range of 280 to 780 nm. The average transmittance was then calculated for three distinct spectral regions: The visible light spectrum (380–780 nm), UVA (316–380 nm), and UVB (280–315 nm).

**Equilibrium water content (EWC) and swelling ratio**

EWC of the lenses was determined using the gravimetric method, in accordance with the ISO 18369-4:2017 standard. For each group, five lenses (*n* = 5) were fully hydrated by immersion in PBS at room temperature for 24 h. After equilibration, each lens was removed from the solution, gently blotted with Kimwipes to remove excess surface moisture, and immediately weighed to obtain the mass of the hydrated lens (*W*_hydrated_). The lenses were subsequently dried in an oven at 100 °C for 24 h to a constant weight, and the mass of the dry lens (*W*_dry_) was recorded. The EWC was then calculated as a percentage using the following equation:

$\mathrm{EWC}\left( \% \right)=\frac{W_{\mathrm{hydrated}}-W_{\mathrm{dry}}}{W_{\mathrm{hydrated}}}\times100\%$	(S3)

The swelling ratio was determined using the same gravimetric method as the EWC. The swelling ratio was then calculated as a percentage using the following equation:

$Swelling ratio \left( \% \right)=\frac{W_{\mathrm{hydrated}}-W_{\mathrm{dry}}}{W_{\mathrm{dry}}}\times100\%$	(S4)

**Mechanical properties**

Prior to testing, the contact lens samples were prepared as dumbbell-shaped specimens with a length of 100 mm and a width of 4 mm to ensure measurement consistency and reproducibility. The mechanical properties of the samples were characterized using a universal testing machine (AI-3000-U, Gotech Testing Machines Inc., Taiwan). Uniaxial tensile tests were conducted at room temperature with an initial gauge length of 3.45 mm and a crosshead speed of 10 mm/min.

**Oxygen permeability (Dk)**

The oxygen permeability (Dk) of the lenses was determined using a polarographic method, based on ISO 18369-4:2017. Measurements were performed using an oxygen polarographic amplifier system (Createch Rehder, Inc., USA), which comprises a 201T oxygen permeometer, a polarographic cell mounting fixture, and an ET-3 thickness gauge. Prior to measurement, five lenses from each sample group were thoroughly soaked in PBS at 35°C overnight to ensure full hydration. The central thickness of each hydrated lens was then measured using the ET-3 thickness gauge. Subsequently, the prepared lens was mounted onto the polarographic cell mounting fixture, and the entire setup was placed within a temperature-humidity chamber maintained at 35 °C and 98% relative humidity (RH). Once mounted, the current signal generated by the permeometer was transmitted to the 201T oxygen permeometer. Measurements were initiated only after the current signal achieved a steady state, at which point the stable current value was recorded. To account for potential variations, this process was repeated using two to five stacked lenses. The preliminary Dk values obtained were corrected for both edge effects and boundary layer effects. Finally, to ensure measurement accuracy and comparability across samples, the corrected Dk values were calibrated against four reference rigid gas-permeable (RGP) lenses: Fluoroperm 30 (Dk = 26.8), Equalens I (Dk = 51.8), Quantum 2 (Dk = 101.0), and Menicon SF-P (Dk = 130.9).

**Long-term stability and durability test**

The long-term stability of the modified lenses was systematically evaluated through four distinct assays: long-term storage, repeated hydration/dehydration cycles, simulated mechanical wear, and accelerated aging tests. Throughout all the aforementioned procedures, the durability of the surface coating was continuously monitored by measuring the static water contact angle.

Long-term storage test: The lenses were individually incubated in double-distilled water, physiological saline (0.9% NaCl), simulated tear fluid (STF), and a protein solution at 35°C for 14 days under continuous shaking. The static contact angles of the samples were recorded on days 1, 7, and 14. The STF was prepared by dissolving 0.67 g of sodium chloride, 0.20 g of sodium bicarbonate, and 0.008 g of calcium chloride dihydrate in double-distilled water to reach a total volume of 100 mL, with the final pH adjusted to 7.4. The protein solution contained 2 mg/mL of bovine serum albumin (BSA) and 2 mg/mL lysozyme.

Repeated hydration/dehydration cycles: The lenses were immersed in STF overnight to ensure complete hydration, then dehydrated in an oven at 100°C overnight. This alternating sequence constituted one complete cycle. A total of five consecutive cycles was performed, and the static contact angle was measured at the conclusion of each cycle.

Simulated mechanical wear: To replicate the mechanical stress generated during human blinking, the lenses were submerged in STF and subjected to 200 cycles of manual rubbing using gloved fingers. The static contact angle was evaluated immediately after the completion of the rubbing procedure.

Accelerated aging test: The samples, fully immersed in STF, underwent three consecutive cycles of steam sterilization in an autoclave. Each sterilization cycle was conducted at 121°C for 20 minutes, after which the static contact angle was measured.

**Cell cytotoxicity**

The *in vitro* cytotoxicity of the contact lenses was evaluated in accordance with ISO 10993-5. Cell culture medium was prepared by mixing 89% minimum essential medium (MEM), 10% fetal bovine serum (FBS), and 1% penicillin–streptomycin (P/S). Lenses were sterilized by exposure to UV light for 30 minutes and subsequently immersed in the prepared cell culture medium. The lens-containing medium was incubated at 37 °C for 24 h to obtain lens extracts. After incubation, the extracts were collected, and L929 (RRID: CVCL_0462) or V79 (RRID: CVCL_2796) cells were seeded into new 24-well culture plates at a density of 1×10^5^ cells/well, and allowed to attach for 24 h in an incubator with 5% CO₂ at 37 °C. The culture medium was then replaced with the lens extract and incubated for an additional 24 h. Following exposure, 0.5 mg/mL MTT reagent was added to each well and incubated for 4 h. The MTT-containing medium was then removed, and the resulting formazan crystals were dissolved in DMSO. The optical density was measured at 595 nm to quantify cell viability.

**BSA adhesion test**

Protein adsorption on contact lens samples was quantified using the BCA™ Protein Assay Kit (Thermo Scientific™) according to the manufacturer's protocol. Lenses were irradiated with UV light for 30 minutes and incubated in a 2 mg/mL bovine serum albumin (BSA) solution at 37 °C for 24 h. After incubation, lenses were rinsed with PBS and transferred to fresh PBS for 2 h at 37 °C to extract adsorbed protein. The protein content of the extract was determined colorimetrically at 562 nm following reaction with BCA reagents. Standard curves were constructed for quantitative analysis, and the amount of protein adsorbed was calculated based on measured absorbance values.

**Lysozyme activity test**

Lysozyme activity adsorbed to contact lens surfaces was quantified using a turbidimetric assay with *Micrococcus lysodeikticus* as substrate (Sigma-Aldrich, L9295). After incubation of lens samples in extraction buffer at 37 °C for 3 h, supernatant was collected and analyzed in a 96-well microplate. For each assay, 190 μL of substrate suspension and 10 μL of sample were combined, and the decrease in absorbance at 492 nm was measured over 5 minutes using a microplate reader. Egg white lysozyme standards (Sigma-Aldrich, L6876; 200–400 units/mL) served as positive controls. Relative lysozyme activity was determined by comparison to standard curves.

**Nuclear Magnetic Resonance (NMR)**

^1^H and ^13^C NMR spectra were recorded on a VNMRS-600 spectrometer (Varian, Inc., USA) operating at 600 MHz for protons and 125 MHz for carbon. All measurements were performed using chloroform-*d* or DMSO-*d*_6_ as the solvent.

**Mass Spectrometry**

High-resolution mass spectra were acquired using a Bruker Impact HD quadrupole time-of-flight (Q-TOF) mass spectrometer (Bruker Corp., USA). The samples were infused into the electrospray ionization (ESI) source operating in positive ion mode. Mass spectra were recorded over the range of *m/z* 200–1500.

# Chemical Synthesis

**Synthesis of Ethyl 4-(4-acetyl-2-methoxyphenoxy)butanoate 1**

To a solution of 4-hydroxy-3-methoxyphenacyl (10.0 g, 60.2 mmol) in anhydrous *N,N*-dimethylformamide (DMF, 100 mL) under a nitrogen atmosphere, potassium carbonate (12.4 g, 89.7 mmol) was added. The resulting suspension was stirred for 30 min prior to the addition of ethyl 4-bromobutyrate (8.60 mL, 60.2 mmol). The reaction mixture was stirred at room temperature (25°C) for 15 h, followed by heating at 50°C for an additional 3 h. Reaction progress was monitored by thin-layer chromatography (TLC; 30% ethyl acetate/hexane). Upon completion, the mixture was poured into deionized water (1.5 L) to dissolve inorganic salts and precipitate the product. After stirring at room temperature for 2 h and subsequent cooling, the precipitate was isolated by vacuum filtration and dried under reduced pressure at 40°C to afford the desired product as a white solid (15.3 g, 91% yield). ^1^H NMR (600 MHz, CDCl_3_, δ): 7.55 (dd, *J* = 8.4, 2.0 Hz, 1H), 7.52 (d, *J* = 2.1 Hz, 1H), 6.89 (d, *J* = 8.4 Hz, 1H), 4.17–4.13 (m, 4H), 3.91 (s, 3H), 2.56 (s, 3H), 2.54 (t, *J* = 7.2 Hz, 2H), 2.21–2.16 (m, 2H), 1.26 (t, *J* = 7.2 Hz, 3H) (**Figure S32**). HRMS (ESI+) *m/z*: [M + Na]^+^ calcd for C_15_H_20_O_5_Na, 303.1203; found, 303.1204.

**Synthesis of Ethyl 4-(4-acetyl-2-methoxy-5-nitrophenoxy)butanoate 2**

Nitric acid (165 mL) was cooled in an ice bath, followed by the slow addition of acetic anhydride (29 mL) while strictly maintaining the temperature below 5°C. A solution of compound **1** (10.0 g, 35.6 mmol) in acetic anhydride (20 mL) was then added dropwise to the precooled nitrating mixture. After stirring at < 5°C for 3 h, the reaction mixture was carefully poured onto a mixture of crushed ice and deionized water. The resulting precipitate was collected by vacuum filtration, washed extensively with deionized water, and air-dried to yield the crude product as a yellow-orange solid. Purification was performed by recrystallization from anhydrous ethanol. Briefly, the crude solid was dissolved in boiling ethanol, filtered hot to remove insoluble impurities, and the filtrate was allowed to cool to room temperature before being stored at 4°C overnight. The resulting crystals were collected by vacuum filtration, washed with deionized water, and dried under reduced pressure to afford the pure product as a yellow solid (8.68 g, 75% yield). ^1^H NMR (600 MHz, CDCl_3_, δ): 7.60 (s, 1H), 6.74 (s, 1H), 4.17–4.13 (m, 4H), 3.94 (s, 3H), 2.53 (t, *J* = 7.2 Hz, 2H), 2.48 (s, 3H), 2.21–2.16 (m, 2H), 1.25 (t, *J* = 7.2 Hz, 3H) (**Figure S33**). HRMS (ESI+) *m/z*: [M + Na]^+^ calcd for C_15_H_19_NO_7_Na, 348.1054; found, 348.1055.

**Synthesis of Ethyl 4-(4-(1-hydroxyethyl)-2-methoxy-5-nitrophenoxy)butanoate 3**

Under a nitrogen atmosphere, compound **2** (10.0 g, 30.7 mmol) was dissolved in anhydrous ethanol (200 mL) with gentle heating at 38°C. The solution was then cooled to 0°C in an ice bath, and sodium borohydride (0.750 g, 19.9 mmol) was added. Following the addition, the reaction mixture was stirred at 38°C overnight, during which the solution transitioned to a deep red color. Reaction completion was confirmed by TLC (30% ethyl acetate/hexane). The mixture was then slowly poured into deionized water (3 L) under vigorous stirring. After 30 min of stirring, the resulting suspension was aged at 4°C overnight to ensure complete precipitation. The precipitate was collected by vacuum filtration, washed with deionized water, and subsequently dissolved in dichloromethane. The organic phase was washed successively with deionized water and brine, dried over anhydrous magnesium sulfate, filtered, and concentrated under reduced pressure. The residue obtained was further dried in vacuum to afford the desired product as a pale yellow solid (8.04 g, 80% yield). ^1^H NMR (600 MHz, DMSO-*d*_6_, δ): 7.52 (s, 1H), 7.35 (s, 1H), 5.48 (d, *J* = 4.2 Hz, 1H), 5.27–5.23 (m, 1H), 4.08–4.05 (m, 4H), 3.90 (s, 3H), 2.45 (t, *J* = 7.2 Hz, 2H), 2.01–1.96 (m, 2H), 1.36 (d, *J* = 6.0 Hz, 3H), 1.18 (t, *J* = 7.2 Hz, 3H) (**Figure S34**). HRMS (ESI+) *m/z*: [M + Na]^+^ calcd for C_15_H_21_NO_7_Na, 350.1210; found, 350.1211.

**Synthesis of 4-(4-(1-hydroxyethyl)-2-methoxy-5-nitrophenoxy)butanoic acid 4**

Finely ground compound **3** (3.0 g, 9.2 mmol) was suspended in deionized water (90 mL), followed by the addition of trifluoroacetic acid (TFA, 10 mL). The reaction mixture was vigorously stirred and heated at 90 °C under reflux for 12 h. After TLC analysis indicated reaction completion, the mixture was cooled to room temperature to induce precipitation. The solid was collected by vacuum filtration, washed with cold deionized water, and subsequently dissolved in ethyl acetate. The organic phase was washed successively with deionized water and brine, dried over anhydrous magnesium sulfate, filtered, and concentrated under reduced pressure. The residue obtained was further dried in vacuum to afford the desired product as a pale yellow solid (1.39 g, 51% yield). ^1^H NMR (600 MHz, DMSO-*d*_6_, δ): 12.18 (s, 1H), 7.53 (s, 1H), 7.36 (s, 1H), 5.49 (s, 1H), 5.27–5.23 (m, 1H), 4.07–4.04 (m, 2H), 3.90 (s, 3H), 2.39 (t, *J* = 7.2 Hz, 2H), 1.97–1.92 (m, 2H), 1.36 (d, *J* = 6.0 Hz, 3H) (**Figure S35**). HRMS (ESI+) *m/z*: [M + Na]^+^ calcd for C_13_H_17_NO_7_Na, 322.0897; found, 322.0897.

**Synthesis of 4-(4-(1-(methacryloyloxy)ethyl)-2-methoxy-5-nitrophenoxy)butanoic acid 5**

Under a nitrogen atmosphere and protected from light, compound **4** (1.0 g, 3.3 mmol) was dissolved in a 2.0 M aqueous sodium hydroxide solution. The mixture was cooled to < 5 °C in an ice bath, followed by the slow addition of methacryloyl chloride (1.0 mL). The reaction was stirred at this temperature for 15 min. Once TLC analysis (90% ethyl acetate in hexanes) indicated complete consumption of the starting material, the mixture was carefully acidified to pH ~2 via the dropwise addition of 1.0 M aqueous hydrochloric acid, which induced the formation of a yellow precipitate. The solid was collected by vacuum filtration, washed with deionized water, and dried in vacuum. The crude material was subsequently purified by silica gel column chromatography (80% ethyl acetate/hexane) to afford the desired product as a slightly sticky yellow solid (0.57 g, 47% yield). ^1^H NMR (600 MHz, DMSO-*d*_6_, δ): 12.17 (s, 1H), 7.57 (s, 1H), 7.15 (s, 1H), 6.27 (q, *J* = 6.6 Hz, 1H), 6.13 (s, 1H), 5.74-5.70 (m, 1H), 4.10–4.04 (m, 2H), 3.90 (s, 3H), 2.38 (t, *J* = 7.2 Hz, 2H), 1.99–1.91 (m, 2H), 1.90-1.86 (m, 3H), 1.62 (d, *J* = 6.6 Hz, 3H) (**Figure S36**). HRMS (ESI+) *m/z*: [M+Na]^+^ calcd for C_17_H_21_NO_8_Na, 390.1159; found, 390.1159.

**Synthesis of 2-((8*S*,9*R*,10*S*,11*S*,13*S*,14*S*,16*R*,17*R*)-9-fluoro-11,17-dihydroxy-10,13,16-trimethyl-3-oxo-6,7,8,9,10,11,12,13,14,15,16,17-dodecahydro-3*H*-cyclopenta[a]phenanthren-17-yl)-2-oxoethyl 4-(4-(1-(methacryloyloxy)ethyl)-2-methoxy-5-nitrophenoxy)butanoate (MA-NB-DXMS)**

Under a nitrogen atmosphere and protected from light, compound **5** (0.36 g, 1.0 mmol) and 4-dimethylaminopyridine (DMAP, 0.061 g, 0.50 mmol) were dissolved in dichloromethane (25 mL). The mixture was cooled to < 5°C in an ice bath. A separate solution of dexamethasone (0.39 g, 1.0 mmol) in tetrahydrofuran (25 mL) was then added to the cooled mixture, followed by the addition of *N*,*N*′-diisopropylcarbodiimide (DIC, 0.16 mL, 1.0 mmol). The reaction mixture was stirred for 12 h, and the reaction progress was monitored by TLC (50% ethyl acetate/hexane). Upon completion, the solvents were removed under reduced pressure. The resulting residue was partitioned between dichloromethane (100 mL) and deionized water (20 mL). The organic layer was separated and concentrated in vacuum. The crude material was purified by silica gel column chromatography (40% ethyl acetate/hexane) to afford the desired product as a sticky yellow solid (0.32 g, 43% yield). ^1^H NMR (600 MHz, DMSO-*d*_6_, δ): 7.58 (s, 1H), 7.29 (d, *J* = 10.2 Hz, 1H), 7.16 (s, 1H), 6.28 (q, *J* = 6.6 Hz, 1H), 6.22 (dd, *J* = 10.2, 1.8 Hz, 1H), 6.14–6.13 (m, 1H), 6.02-5.99 (m, 1H), 5.75–5.70 (m, 1H), 5.41–5.37 (m, 1H), 5.16 (s, 1H), 5.05 (dd, *J* = 17.4, 3.0 Hz, 1H), 4.83 (dd, *J* = 17.4, 1.8 Hz, 1H), 4.17–4.11 (m, 3H), 3.91 (s, 3H), 2.91–2.84 (m, 1H), 2.64–2.59 (m, 1H), 2.56 (t, *J* = 7.5 Hz, 2H), 2.40–2.30 (m, 2H), 2.22–2.15 (m, 2H),2.06–2.01 (m, 2H), 1.89–1.88 (m, 3H), 1.78–1.75 (m, 1H), 1.63 (d, *J* = 6.6 Hz, 3H), 1.59–1.57 (m, 1H), 1.49 (s, 3H), 1.36–1.33 (m, 1H), 1.09–1.05 (m, 1H), 1.00 (d, *J* = 6.6 Hz, 1H), 0.88 (d, *J* = 3.3 Hz, 3H), 0.79 (d, *J* = 7.2 Hz, 3H) (**Figure S37**); ^13^C NMR (125 MHz, DMSO-*d*_6_, δ, ^1^H and ^19^F decoupling): 204.9, 185.3, 171.9, 167.1, 165.7, 156.8, 153.5, 152.7, 146.9, 139.7, 135.7, 131.8, 129.0, 126.3, 124.1, 108.8, 108.5, 101.3, 90.5, 70.5, 68.1, 67.9, 67.7, 56.2, 48.0, 43.3, 35.6, 35.4, 33.6, 31.9, 30.3, 29.8, 27.3, 24.1, 23.3, 21.3, 17.9, 16.2, 15.1. HRMS (ESI+) *m/z*: [M+H]^+^ calcd for C_39_H_49_FNO_12_, 742.3233; found, 742.3231 (**Figure S38**).

**Table S1.** Atomic concentrations of the pristine and HEMA-modified contact lenses determined via XPS.

| Sample | C (%) | | O (%) | | N (%) | Si (%) |
| --- | --- | --- | --- | --- | --- | --- |
| Acuvue | 62.99 ± 1.75% | 21.21 ± 0.88% | | 3.33 ± 1.46% | | 12.47 ± 1.36% |
| Acuvue-10HEMA | 65.70 ± 0.30% | 26.74 ± 1.74% | | 1.46 ± 0.71% | | 6.09 ± 1.19% |
| Acuvue-20HEMA | 65.24 ± 0.33% | 28.15 ± 0.56% | | 0.85 ± 0.09% | | 5.76 ± 0.84% |
| Clariti | 67.98 ± 0.08% | 18.44 ± 0.13% | | 4.32 ± 0.23% | | 9.26 ± 0.34% |
| Clariti-10HEMA | 68.02 ± 0.41% | 19.17 ± 0.03% | | 4.71 ± 0.13% | | 8.10 ± 0.53% |
| Clariti-20HEMA | 65.39 ± 0.29% | 23.37 ± 0.76% | | 3.09 ± 0.55% | | 8.15 ± 0.39% |
| Ultra | 65.98 ± 1.18% | 18.43 ± 0.99% | | 4.58 ± 0.47% | | 11.01 ± 0.66% |
| Ultra-10HEMA | 67.09 ± 0.45% | 20.27 ± 0.47% | | 3.23 ± 0.25% | | 9.45 ± 0.33% |
| Ultra-20HEMA | 67.46 ± 1.08% | 22.33 ± 1.35% | | 2.80 ± 0.30% | | 7.42 ± 0.64% |

**Table S2.** Quantitative nanomechanical properties and layer thicknesses derived from AFM force–distance analysis. The table summarizes the reduced modulus (*E*′) and Young’s modulus (*E*) for both the base lens substrate and the outer poly(HEMA) coating. It also details the calculated thicknesses of the discrete coating layer (*t*_coating_) and the semi-IPN structure (*t*_semi-IPN_) across different HEMA concentrations (2.5%, 10%, and 20%). All parameters were extracted using the self-consistent modulus algorithms described in the Methods. Data are presented as mean ± s.e.m. (*n* = 3).

| Sample | Base Lens | | Coating | | Thickness | |
| --- | --- | --- | --- | --- | --- | --- |
|  | *E′* (MPa) | *E* (MPa) | *E′* (MPa) | *E* (MPa) | *t*_coating_ (nm) | *t*_semi-IPN_ (nm) |
| Acuvue | 2.22 ± 0.45 | 1.67 ± 0.34 | – | – | – | – |
| 2.5HEMA | 1.09 ± 0.09 | 0.82 ± 0.07 | 0.26 ± 0.11 | 0.19 ± 0.08 | 19.23 ± 1.10 | – |
| 10HEMA | 0.81 ± 0.13 | 0.61 ± 0.10 | 0.10 ± 0.02 | 0.08 ± 0.02 | 33.75 ± 2.71 | 25.18 ± 2.74 |
| 20HEMA | 1.02 ± 0.02 | 0.77 ± 0.01 | 0.06 ± 0.01 | 0.04 ± 0.01 | 46.28 ± 0.61 | 70.26 ± 1.60 |
| Clariti | 3.75 ± 0.26 | 2.82 ± 0.19 | – | – | – | – |
| 2.5HEMA | 1.57 ± 0.07 | 1.18 ± 0.05 | 0.18 ± 0.03 | 0.13 ± 0.02 | 36.83 ± 5.31 | – |
| 10HEMA | 2.11 ± 0.14 | 1.58 ± 0.10 | 0.07 ± 0.01 | 0.06 ± 0.01 | 46.22 ± 3.66 | 25.44 ± 3.65 |
| 20HEMA | 1.25 ± 0.13 | 0.94 ± 0.10 | 0.09 ± 0.01 | 0.07 ± 0.01 | 71.68 ± 6.74 | 74.39 ± 5.70 |
| Ultra | 0.78 ± 0.01 | 0.59 ± 0.01 | – | – | – | – |
| 2.5HEMA | 0.82 ± 0.04 | 0.62 ± 0.03 | 0.14 ± 0.04 | 0.10 ± 0.03 | 39.60 ± 6.47 | – |
| 10HEMA | 0.98 ± 0.03 | 0.73 ± 0.02 | 0.13 ± 0.03 | 0.10 ± 0.02 | 36.74 ± 0.58 | 22.62 ± 2.20 |
| 20HEMA | 0.67 ± 0.04 | 0.50 ± 0.03 | 0.04 ± 0.01 | 0.03 ± 0.01 | 54.31 ± 2.00 | 67.38 ± 3.93 |

**Table S3.** Optical transmittance and UV-blocking classification of pristine and surface-modified lenses. The table presents the quantitative transmittance values for visible light (380–780 nm), UVA (316–380 nm), and UVB (280–315 nm) across pristine, HEMA-modified, and drug-functionalized (MA-NB-DXMS and HEMA/MA-NB-DXMS) lenses. UV blocking categories are assigned in accordance with ANSI Z80.20 standards: Class I requires UVA < 10% and UVB < 1% (satisfied by Acuvue variants), while Class II requires UVA < 50% and UVB < 5% (satisfied by Clariti and Ultra variants). Data are presented as mean ± s.e.m. (*n* = 3).

|  | Transmittance | | | | |  |
| --- | --- | --- | --- | --- | --- | --- |
| Sample | Visible  (380nm–780nm) | | UVA  (316nm–380nm) | | UVB  (280nm–315nm) | UV Blocker |
| Acuvue | 96.71 ± 0.48% | 3.02 ± 0.21% | | 0.04 ± 0.01% | | UV Class Ⅰ |
| Acuvue-10HEMA | 97.70 ± 0.20% | 3.39 ± 0.13% | | 0.04 ± 0.01% | |  |
| Acuvue-20HEMA | 97.15 ± 0.24% | 4.00 ± 0.05% | | 0.04 ± 0.01% | |  |
| Clariti | 97.75 ± 0.27% | 24.45 ± 0.29% | | 1.07 ± 0.05% | | UV Class Ⅱ |
| Clariti-10HEMA | 97.72 ± 0.13% | 20.28 ± 0.17% | | 0.39 ± 0.03% | |  |
| Clariti-20HEMA | 98.11 ± 0.15% | 24.76 ± 0.32% | | 1.11 ± 0.07% | |  |
| Ultra | 95.06 ± 0.19% | 21.32 ± 0.20% | | 0.56 ± 0.02% | | UV Class Ⅱ |
| Ultra-10HEMA | 95.02 ± 0.19% | 22.10 ± 0.09% | | 0.67 ± 0.02% | |  |
| Ultra-20HEMA | 94.01 ± 0.30% | 21.35 ± 2.69% | | 0.77 ± 0.51% | |  |
| Acuvue MA-NB-DXMS | 91.60 ± 0.44% | 1.09 ± 0.10% | | 0.03 ± 0.01% | | UV Class Ⅰ |
| Acuvue HEMA/MA-NB-DXMS | 95.98 ± 0.19% | 2.02 ± 0.31% | | 0.04 ± 0.01% | |  |

**Figure S1.** ATR-FTIR spectra (500–2,000 cm^-1^) (**a**) and (400–4,000 cm^-1^) (**b**) of pure poly(HEMA) (reference, black) compared with pristine and HEMA-modified (10% and 20% HEMA) Acuvue (blue), Clariti (green), and Ultra (orange) lenses. Gray shaded regions highlight the C-O-C (1,158 cm^-1^) and C=O (1,720 cm^-1^) vibrations.

**Figure S2.** XPS survey spectra displaying the surface elemental composition of pristine and HEMA-modified (10% and 20% HEMA) Acuvue (blue), Clariti (green), and Ultra (orange) lenses. Key characteristic peaks (C1s, N1s, O1s, Si2p) are labeled.

**Figure S3.** Atomic concentrations of the pristine and HEMA-modified contact lenses determined via XPS.

**Figure S4.** High-resolution XPS C1s spectra of Acuvue-10HEMA (**a**), pristine and HEMA-modified (10% and 20% HEMA) Clariti (**b**) and Ultra (**c**) lenses.

**Figure S5.** High-resolution XPS O 1s spectra of Acuvue and Acuvue-20HEMA. Precise deconvolution of the O 1s spectra for this complex polymer network is extremely difficult. The expected binding energies for these oxygen-containing species, specifically N-C=O (~531.3 eV), O-C=O/Si-O-Si (~532.3 eV), and C-O-C/C-OH (~533.3 eV), are extremely close to one another, separated by only about 1.0 eV. This subtle chemical shift results in severe spectral overlapping within the O 1s envelope.

**Figure S6.** Evolution of high-resolution C1s spectra for Clariti-20HEMA and Ultra-20HEMA at progressive GCIB sputtering depths. The waterfall plots illustrate the gradual attenuation of the ester signal from the surface to the inner lens (5 min sputtering intervals).

**Figure S7.** Depth-dependent poly(HEMA) coverage profiles during GCIB sputtering (0–30 min).

**Figure S8.** Representative cantilever deflection curves and linearized force–indentation plots for HEMA-modified (2.5% and 10% HEMA) Acuvue lenses. The true surface contact point (red dot) and the coating/semi-IPN interface (blue dot) were identified using the self-consistent modulus algorithm. These key points demarcate three distinct mechanical regimes: the soft outer poly(HEMA) coating layer (red shaded region), the intermediate semi-IPN structure (blue shaded region), and the underlying base lens (gray shaded region).

**Figure S9.** Representative cantilever deflection curves and linearized force–indentation plots for pristine and HEMA-modified (2.5%, 10%, and 20% HEMA) Clariti lenses. The true surface contact point (red dot) and the coating/semi-IPN interface (blue dot) were identified using the self-consistent modulus algorithm. These key points demarcate three distinct mechanical regimes: the soft outer poly(HEMA) coating layer (red shaded region), the intermediate semi-IPN structure (blue shaded region), and the underlying base lens (gray shaded region).

**Figure S10.** Representative cantilever deflection curves and linearized force–indentation plots for pristine and HEMA-modified (2.5%, 10%, and 20% HEMA) Ultra lenses. The true surface contact point (red dot) and the coating/semi-IPN interface (blue dot) were identified using the self-consistent modulus algorithm. These key points demarcate three distinct mechanical regimes: the soft outer poly(HEMA) coating layer (red shaded region), the intermediate semi-IPN structure (blue shaded region), and the underlying base lens (gray shaded region).

**Figure S11.** Long-term stability and durability of HEMA-modified contact lenses. (**a–d**) Evolution of static contact angles following storage in aqueous media (**a**), physiological saline (**b**), simulated tear fluid (**c**), and protein solutions (**d**). (**e**) Static contact angles recorded across 5 continuous hydration/dehydration cycles. (**f**) Static contact angles assessed after manual rubbing to simulate the mechanical wear of blinking. (**g**) Static contact angles determined after accelerated aging through three cycles of high-temperature steam sterilization. Data are presented as mean ± s.e.m. (*n* = 3). Statistical significance was calculated using one-way ANOVA: ns, not significant (*P* > 0.05), **P* < 0.05, ****P* < 0.001, and *****P* < 0.0001.

**Figure S12.** Stability assessment via accelerated aging. ATR-FTIR spectra (500–2,000 cm^-1^) comparing pristine lenses with HEMA-modified (20% HEMA) lenses after accelerated aging. The gray shaded regions highlight the persistent characteristic peaks of C-O-C (1,158 cm^-1^) and C=O (1,720 cm^-1^). The retention of these spectral features after aging confirms the long-term stability of the poly(HEMA) coating on the lens surface.

**Figure S13.** Dynamic contact angles of pristine and HEMA-modified Acuvue contact lenses. (**a**, **b**) Representative optical images of the receding (**a**) and advancing (**b**) contact angles for the pristine Acuvue lenses. (**c**, **d**) Corresponding optical images demonstrating the receding (**c**) and advancing (**d**) contact angles for the Acuvue-20HEMA lenses.

**Figure S14.** Characteristic ToF-SIMS mass spectra of pristine and modified lens surfaces. Representative positive-ion mass spectra acquired from the surface of (**a**) pristine Acuvue and (**b**) Acuvue-20HEMA lenses. The spectra highlight the diagnostic fragment ions utilized for chemical mapping and depth profiling. HEMA-specific markers (red dots) include CH_3_O^+^, C_2_H_5_O^+^, C_4_H_5_O^+^, C_6_H_9_O_2_^+^, and C_6_H_11_O_2_^+^. Siloxane-specific substrate markers (blue dots) include C_3_H_9_Si^+^ and C_5_H_15_OSi_2_^+^.

**Figure S15.** Characteristic ToF-SIMS mass spectra of pristine and modified lens surfaces. Representative positive-ion mass spectra acquired from the surface of (**a**) pristine Clariti and (**b**) Clariti -20HEMA lenses. The spectra highlight the diagnostic fragment ions utilized for chemical mapping and depth profiling. HEMA-specific markers (red dots) include CH_3_O^+^, C_2_H_5_O^+^, C_4_H_5_O^+^, C_6_H_9_O_2_^+^, and C_6_H_11_O_2_^+^. Siloxane-specific substrate markers (blue dots) include C_3_H_9_Si^+^ and C_5_H_15_OSi_2_^+^.

**Figure S16.** Characteristic ToF-SIMS mass spectra of pristine and modified lens surfaces. Representative positive-ion mass spectra acquired from the surface of (**a**) pristine Ultra and (**b**) Ultra-20HEMA lenses. The spectra highlight the diagnostic fragment ions utilized for chemical mapping and depth profiling. HEMA-specific markers (red dots) include CH_3_O^+^, C_2_H_5_O^+^, C_4_H_5_O^+^, C_6_H_9_O_2_^+^, and C_6_H_11_O_2_^+^. Siloxane-specific substrate markers (blue dots) include C_3_H_9_Si^+^ and C_5_H_15_OSi_2_^+^.

**Figure S17.** ToF-SIMS characterization of HEMA-modified contact lenses. (**a, b**) Depth profiles acquired using GCIB sputtering for pristine Clariti (**a**) and Clariti-20HEMA (**b**) lenses. (**c, d**) Three-dimensional reconstructed images (100 × 100 µm^2^) of pristine Clariti (**c**) and Clariti-20HEMA (**d**) lenses. The panels display the spatial distribution of the HEMA-specific fragment C_2_H_5_O^+^ (red), the siloxane-specific fragment C_3_H_9_Si^+^ (blue), and their overlay. The vertical axis corresponds to a total sputtering time of 300 s. (**e, f**) Lateral overlap chemical maps comparing Clariti and Clariti-20HEMA at surface (**e**) and a sputtering time of 20 s (**f**). Red and blue pixels represent C_2_H_5_O^+^ and C_3_H_9_Si^+^ signals, respectively. Scale bar, 20 µm.

**Figure S18.** ToF-SIMS characterization of HEMA-modified contact lenses. (**a, b**) Depth profiles acquired using GCIB sputtering for pristine Ultra (**a**) and Ultra-20HEMA (**b**) lenses. (**c, d**) Three-dimensional reconstructed images (100 × 100 µm^2^) of pristine Ultra (**c**) and Ultra -20HEMA (**d**) lenses. The panels display the spatial distribution of the HEMA-specific fragment C_2_H_5_O^+^ (red), the siloxane-specific fragment C_3_H_9_Si^+^ (blue), and their overlay. The vertical axis corresponds to a total sputtering time of 300 s. (**e, f**) Lateral overlap chemical maps comparing Clariti and Clariti-20HEMA at surface (**e**) and a sputtering time of 20 s (**f**). Red and blue pixels represent C_2_H_5_O^+^ and C_3_H_9_Si^+^ signals, respectively. Scale bar, 20 µm.

**Figure S19.** Representative AFM topography images comparing pristine and HEMA-modified (20% HEMA) surfaces for Clariti and Ultra lenses. The images reveal the surface morphology before and after the formation of the poly(HEMA) coating layer. The vertical color scale indicates a height range from -20 nm to +20 nm. Scale bars, 1 µm.

**Figure S20.** In vitro biocompatibility assessment of HEMA-modified lenses using L929 (**a**) and V79 (**b**) fibroblasts. Quantitative analysis of cell viability measured by cytotoxicity assays for pristine and HEMA-modified (10% and 20% HEMA) Acuvue (blue), Clariti (green), and Ultra (orange) lenses. The study includes a medium control, a positive control (RMB, cytotoxic), and a negative control (RMC, non-toxic) to validate the assay sensitivity. All data are presented as the mean ± s.e.m. (*n* = 3).

**Figure S21.** Protein antifouling performance of HEMA-modified contact lenses. Quantitative analysis of BSA adsorption (**a**) and relative enzymatic activity of lysozyme (**b**) on the lens surfaces. Data are presented as mean ± s.e.m. (*n* = 3). Statistical significance was calculated using one-way ANOVA: ns, not significant (*P* > 0.05), **P* < 0.05, ***P* < 0.01, ****P* < 0.001, and *****P* < 0.0001.

**Figure S22.** Cell antifouling performance of HEMA-modified contact lenses. Adhesion ratio of L929 cells on the lens surfaces. Data are presented as mean ± s.e.m. (*n* = 3). Statistical significance was calculated using one-way ANOVA: **P* < 0.05, ****P* < 0.001, *****P* < 0.0001.

**Figure S23.** Optical transmission spectra of HEMA-modified contact lenses. (**a-c**) Optical transmittance spectra recorded in the 280–780 nm range for pristine and HEMA-modified (10% and 20% HEMA) lenses: Acuvue (**a**), Clariti (**b**), and Ultra (**c**). The shaded regions correspond to the UVB (dark gray, 280–315 nm) and UVA (light gray, 316–380 nm) bands.

**Figure S24.** Calibration of oxygen permeability measurements using reference RGP lenses. Oxygen permeability (Dk) values were calibrated using four certified rigid gas permeable (RGP) reference lenses: Fluoroperm 30 (Dk = 26.8), Equalens I (Dk = 51.8), Quantum 2 (Dk = 101.0), and Menicon SF-P (Dk = 130.9). The left panel displays the linear regression line established by these standards across the full measurement range, while the right panel provides a magnified view of the experimental data for pristine and HEMA-modified (10% and 20%HEMA) Acuvue (blue square), Clariti (green triangle), and Ultra (orange diamond) lenses. The measured Dk values were adjusted according to this calibration curve to eliminate instrumental and environmental offsets. All data (except reference RGPs) are presented as mean ± s.e.m. (*n* = 3).

**Figure S25.** Tensile strength of pristine and HEMA-modified contact lenses. Data are presented as mean ± s.e.m. (*n* = 3). Statistical significance was calculated using ANOVA: ns, not significant (*P* > 0.05).

**Figure S26.** Determination of intrinsic oxygen permeability (Dk) via the multi-lens stacking method. Dk values were derived by plotting the inverse of oxygen transmissibility (*t* Dk^-1^) against the total lens thickness (*t*). Measurements were performed on single and stacked lenses (1 to 5 lenses) to account for boundary layer resistance, in accordance with ISO 18369-4 standards. The plots display the linear regression profiles for pristine and HEMA-modified (10% and 20% HEMA) Acuvue (blue), Clariti (green), and Ultra (orange) lenses. The intrinsic Dk is calculated from the inverse of the slope of the fitted lines.

**Figure S27.** Thermal stability and structural integrity of the photolabile prodrug. (**a**) HPLC chromatograms of the MA-NB-DXMS prodrug monomer measured before (blue) and after (orange) thermal sterilization. The samples were analyzed before (0 min, dark lines) and after (5 min, light lines) exposure to 365 nm UV light (10 mW/cm²). (**b-e**) High-resolution mass spectra corresponding to the characteristic HPLC peaks. (**b, c**) Non-sterilized samples: intact prodrug at 14.3 min (**b**, [M + H]^+^ = 742.3234) and photocleaved product at 11.5 min (**c**, [M + Na]^+^ = 664.2528). (**d, e**) Sterilized samples: intact prodrug at 14.3 min (**d**, [M + H]^+^ = 742.3269) and photocleaved product at 11.5 min (**e**, [M + H]^+^ = 642.2689).

**Figure S28.** XPS characterization of drug-loaded contact lenses. High-resolution F1s spectra of pristine Acuvue, drug-loaded lens (Acuvue MA-NB-DXMS), and the HEMA-modified drug-loaded lens (Acuvue HEMA/MA-NB-DXMS). The F1s signal (685 eV) confirms the successful incorporation of the fluorine-containing drug (dexamethasone).

**Figure S29.** HPLC verification of photocleavage kinetics and calibration for drug quantification. (**a**) Chromatographic monitoring of the MA-NB-DXMS prodrug monomer under 365 nm UV exposure (10 mW/cm²). The shift in retention time from 14.3 min (intact prodrug) to 11.5 min (cleaved drug) confirms efficient photocleavage. (**b**) Standard calibration curve constructed by plotting the peak area of the cleaved product (retention time ≈ 11.5 min) against concentration (0–1 mM) following complete photolysis. The linear regression allows for the precise quantification of released dexamethasone. (**c, d**) Representative HPLC chromatograms of release media collected from Acuvue MA-NB-DXMS (**c**) and Acuvue HEMA/MA-NB-DXMS (**d**) lenses during alternating light on/off cycles (405 nm, 10 mW/cm²). Data in **b** are presented as mean ± s.e.m. (*n* = 3).

**Figure S30.** Optical transmittance spectra comparing the pristine Acuvue lens (black) with the drug-loaded lens (Acuvue MA-NB-DXMS, blue) and the HEMA-modified drug-loaded lens (Acuvue HEMA/MA-NB-DXMS, orange).

**Figure S31.** Optical transmittance spectra comparing the pristine Acuvue lens (black) and the HEMA-modified drug-loaded lens prepared with a high-concentration formulation (Acuvue 0.5% MA-NB-DXMS/0.1% HEMA, red).

**Figure S32.** ¹H NMR spectrum of compound **1** in chloroform-*d*.

**Figure S33.** ¹H NMR spectrum of compound **2** in chloroform-*d*.

**Figure S34.** ¹H NMR spectrum of compound **3** in DMSO-*d*_6_.

**Figure S35.** ¹H NMR spectrum of compound **4** in DMSO-*d*_6_.

**Figure S36.** ¹H NMR spectrum of compound **5** in DMSO-*d*_6_.

**Figure S37.** ¹H NMR spectrum of MA-NB-DXMS in DMSO-*d*_6_.

**Figure S38.** HR-ESI-MS spectrum of MA-NB-DXMS.

# Reference

1. X. Deng, M. Korogiannaki, B. Rastegari, et al. “"Click" Chemistry-Tethered Hyaluronic Acid-Based Contact Lens Coatings Improve Lens Wettability and Lower Protein Adsorption,” *ACS Applied Materials & Interfaces* 8, no. 34 (2016): 22064–22073. <https://doi.org/10.1021/acsami.6b07433>

2. M. Korogiannaki, L. Jones, and H. Sheardown. “Impact of a Hyaluronic Acid-Grafted Layer on the Surface Properties of Model Silicone Hydrogel Contact Lenses,” *Langmuir* 35, no. 4 (2019): 950–961. <https://doi.org/10.1021/acs.langmuir.8b01693>

3. L. Xu, P. Ma, B. Yuan, et al. “Anti-biofouling contact lenses bearing surface-immobilized layers of zwitterionic polymer by one-step modification,” *RSC Advances* 4, no. 29 (2014): 15030–15035. <https://doi.org/10.1039/c3ra47119e>

4. A. M. Kloxin, M. W. Tibbitt, and K. S. Anseth. “Synthesis of photodegradable hydrogels as dynamically tunable cell culture platforms,” *Nature Protocols* 5, no. 12 (2010): 1867–1887. <https://doi.org/10.1038/nprot.2010.139>

5. J. T. Jacob, J. Levet, Jr., T. A. Edwards, N. Dassanayake, and H. Ketelson. “Visualizing hydrophobic domains in silicone hydrogel lenses with Sudan IV,” *Investigative Ophthalmology & Visual Science* 53, no. 7 (2012): 3473–3480. <https://doi.org/10.1167/iovs.11-9104>
